# Increased [^18^F]FDG uptake in the infarcted myocardial area displayed by combined PET/CMR correlates with snRNA-seq-detected inflammatory cell invasion

**DOI:** 10.1007/s00395-024-01064-y

**Published:** 2024-06-26

**Authors:** Dominika Lukovic, Mariann Gyöngyösi, Imre J. Pavo, Julia Mester-Tonczar, Patrick Einzinger, Katrin Zlabinger, Nina Kastner, Andreas Spannbauer, Denise Traxler, Noemi Pavo, Georg Goliasch, Dietmar Pils, Andras Jakab, Zsuzsanna Szankai, Ina Michel-Behnke, Lu Zhang, Yvan Devaux, Senta Graf, Dietrich Beitzke, Johannes Winkler

**Affiliations:** 1 Department of Internal Medicine II, Division of Cardiology, Medical University of Vienna, Vienna, Austria; 2 Division of Pediatric Cardiology, Department of Pediatrics and Adolescent Medicine, Medical University of Vienna, Vienna, Austria; 3 Institute of Information Systems Engineering, Research Unit of Information and Software Engineering, Vienna University of Technology, 1040 Vienna, Austria; 4 Division of General Surgery, Department of Surgery, Comprehensive Cancer Center Vienna, Medical University of Vienna, Vienna, Austria; 5 Center for MR-Research, University Children’s Hospital Zurich, Zurich, Switzerland; 6 Department of Neurology, Kantonsspital Aarau, Aarau, Switzerland; 7 Cardiovascular Research Unit, Department of Population Health, Luxembourg Institute of Health, Strassen, Luxembourg; 8 Department of Biomedical Imaging and Image-Guided Therapy, Medical University of Vienna, Vienna, Austria

**Keywords:** [^18^F]FDG, Acute myocardial infarction, Cardiac PET–MRI imaging, Innate immune response, RNA sequencing

## Abstract

Combined [^18^F]FDG PET-cardiac MRI imaging (PET/CMR) is a useful tool to assess myocardial viability and cardiac function in patients with acute myocardial infarction (AMI). Here, we evaluated the prognostic value of PET/CMR in a porcine closed-chest reperfused AMI (rAMI) model. Late gadolinium enhancement by PET/CMR imaging displayed tracer uptake defect at the infarction site by 3 days after the rAMI in the majority of the animals (group Match, *n* = 28). Increased [^18^F]FDG uptake at the infarcted area (metabolism/contractility mismatch) with reduced tracer uptake in the remote viable myocardium (group Mismatch, *n* = 12) 3 days after rAMI was observed in the animals with larger infarct size and worse left ventricular ejection fraction (LVEF) (34 ± 8.7 vs 42.0 ± 5.2%), with lower LVEF also at the 1-month follow-up (35.8 ± 9.5 vs 43.0 ± 6.3%). Transcriptome analyses by bulk and single-nuclei RNA sequencing of the infarcted myocardium and border zones (*n* = 3 of each group, and 3 sham-operated controls) revealed a strong inflammatory response with infiltration of monocytes and macrophages in the infarcted and border areas in Mismatch animals. Our data indicate a high prognostic relevance of combined PET/MRI in the subacute phase of rAMI for subsequent impairment of heart function and underline the adverse effects of an excessive activation of the innate immune system in the initial phase after rAMI.

**Supplementary Information:**

The online version contains supplementary material available at 10.1007/s00395-024-01064-y.

## Introduction

Adequate estimation of viable myocardium in the subacute phase of myocardial infarction (MI) is of high scientific and clinical importance [12]. Early determination of prognostic indicators for subsequent adverse cardiac remodeling, resulting in heart failure, is a necessity for selection of optimal treatment. Recent progress in medical imaging technologies has enabled the routine use of combined cardiac magnetic resonance imaging (CMR) and positron emission tomography (PET) imaging as a diagnostic tool for acute myocardial infarction (AMI) to assess residual myocardial viability, cardiac function, and infarct [26, 37, 41]. Both technologies have distinct virtues in diagnosis of cardiac pathologies, but also give synergistic information [37].

CMR has become the standard of reference for assessing left and right ventricular function, and tissue characterization. In MI, application of gadolinium contrast agents results in delayed enhancement as disrupted myocyte membranes allow diffusion of extracellular gadolinium into previously intracellular space [61]. Late gadolinium enhancement (LGE) is highly correlated with myocardial necrosis in histological evaluation [61]. CMR is able to differentiate between myocardial edema, necrosis, microvascular obstruction (MVO), or intramyocardial hemorrhage as a potential indicator for reperfusion injury with additional prognostic impact post-AMI [7].

PET imaging is ideally suited for absolute quantification of myocardial perfusion, coronary flow reserve, and visualization and quantification of molecular processes, including inflammation and cell metabolism [32]. Myocardial metabolism and cell viability can be imaged with the radiotracer 2-[^18^F]fluoro-2-deoxy-D-glucose (FDG). Generally, preserved or increased glucose utilization and subsequent [^18^F]FDG uptake is regarded as a measure for cell survival with preserved metabolism, while reduced [^18^F]FDG uptake indicates an area of scarred myocardium [20]. Integrated PET/MRI imaging has been available for a decade [48], but its high cost and complex application defers a more widespread use in clinical cardiology workflows [38]. It is increasingly being used in clinics for synergistic viability and inflammatory focus imaging. On the other hand, myocardial perfusion imaging has largely remained a tool for research [48].

For assessment of myocardial viability, there is generally moderate to good agreement between increased transmurality in CMR with LGE and reduced [^18^F]FDG uptake measured by PET [3]. However, FDG uptake is reflecting glucose metabolism and, in some cases, fails to correlate with viable myocardium, e.g., in case of a mismatch between perfusion and metabolism (hibernation), or when inflammatory cells are recruited to the ischemic area.

An infarcted area is characterized by a reduced number of viable myocytes and generally displays lower uptake of the viability marker [^18^F]FDG uptake [13, 23, 35]. In contrast, in a series of pig reperfused AMI (rAMI) experiments, we detected conflicting signals in the combined PET/CMR imaging in the infarcted area in a number of cases 3 days post-infarct. In these cases, surprisingly high [^18^F]FDG uptake was observed in the infarcted areas and corroborated by increased LGE and reduced local wall motion according to CMR (metabolism/contractility mismatch). Intriguingly, we have found that this metabolism/contractility mismatch subsequently results in significantly stronger loss of cardiac function 1 month post-ischemic injury. We hypothesized that enhanced [^18^F]FDG uptake in ischemic areas in the subacute phase of rAMI might be caused by enhanced inflammatory mechanisms with higher demand and consummation of local glucose ([^18^F]FDG) by the recruited inflammatory cells [53], and pathologic redistribution of the glucose from the non-ischemic viable to the ischemic area. It has been shown that inflammation plays a critical role in determining AMI size and adverse LV remodeling [42]. Another explanation of the apparent metabolism/contractility mismatch might be the imbalance of the energy metabolism between fatty acid oxidation and glucose oxidation, the latter of which has better energy efficiency and utilizes a higher amount of adenosine triphosphate for energy supply in a critical ischemic situation [30]. For investigating the responsible underlying molecular and cellular mechanisms, we conducted a transcriptomic analysis of both bulk and single-nuclei RNA sequencing (snRNA-seq).

## Materials and methods

### Study design

**Fig. 1:**
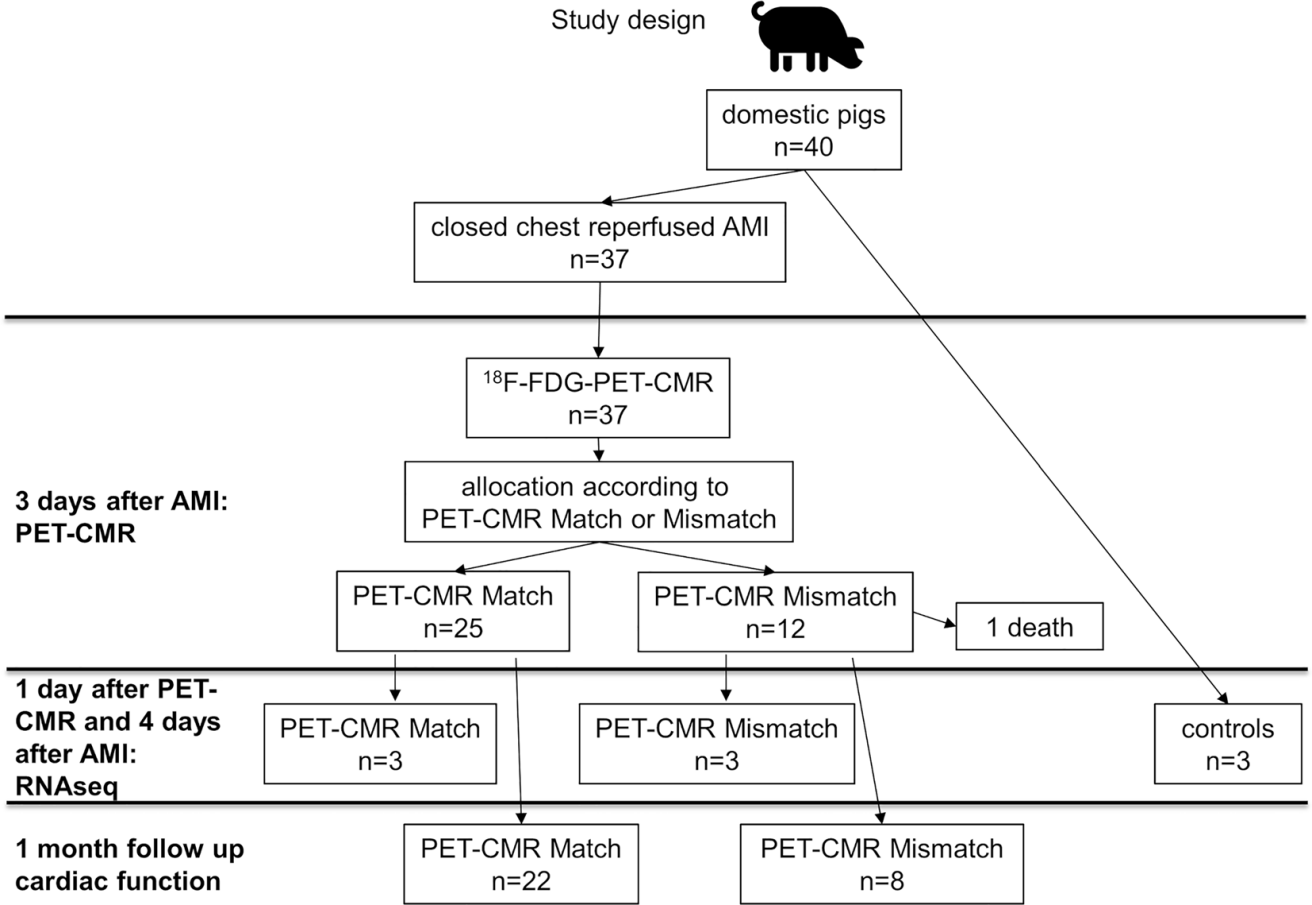
Study design. The flow chart indicates the respective number of animals (*n*) for each group and analysis time point. In total, 37 pigs underwent percutaneous induction of AMI, of which 25 and 12 were allocated to the Match and Mismatch groups, respectively, according to PET–MRI imaging data 3 days post-AMI. Three animals of each group were sacrificed 1 day after the initial PET–MRI imaging for assessing histology, RNA sequencing, and biochemical analysis. The remaining animals were followed up for 1 month for assessment of functional cardiac parameters by PET–MRI imaging

Thirty pigs with 1-month FUP were initially included into the cardiac functional study (Fig. 1). To enable the investigation of the 3-day PET/CMR metabolism/contractility mismatch at the cellular and molecular level, we have included seven additional pigs with rAMI (one died during AMI procedure), and harvested tissues at day four (1 day after PET/MRI due to radiation protection reason) for the myocardial sampling, bulk RNA-seq, and snRNA-seq (Fig. 1).

Under general anesthesia, closed-chest rAMI was induced in 37 domestic pigs by 90 min occlusion of the mid left anterior descending artery with a percutaneous intracoronary balloon, followed by reperfusion. One animal died during the AMI phase. Three days and 1 month after rAMI, respectively, control coronary angiography was performed to ensure the patency of the infarct-related artery, followed by [^18^F]FDG PET–CMR using standardized acquisition protocols (*n* = 30, Match *n* = 22, Mismatch *n* = 8). During the invasive catheterization procedure after a 12-h fasting period, the pigs received 150 mg amiodarone in 500 mL 5% glucose solution, which corresponds to the weight-adjusted intravenous glucose load protocol. TIMI (thrombolysis in myocardial infarction) flow and myocardial blush (contrast density) grade, an angiographic assessment of myocardial reperfusion after acute infarction, was assessed 5 min after reperfusion induction, in accordance to published protocols [16, 54, 57]. TIMI flow was graded from 0 (no flow) to 3 (unlimited distal flow). Myocardial blush was graded as follows: 0 (no myocardial blush) 1, (minimal myocardial blush); 2 (moderate myocardial blush), and 3 (normal myocardial blush). Cardiac functional parameters such as ejection fraction (EF), end-diastolic volume (EDV), infarct size, and mean tracer uptake of the infarcted area were quantitatively assessed. Six animals (three of each the Match and Mismatch groups) were euthanized 1 day after the 3-day [^18^F]FDG PET–CMR imaging to investigate differences in gene expression patterns between animals with and without viability and infarct location mismatch using RNA sequencing and pathway network analyses.

The hearts were explanted and separated into infarct (AMI) and border zones of the left ventricle (LV). The tissue samples were cut into small pieces and submersed in RNA later (frozen for RNA analysis), or in formalin (for histology). Three additional animals of the same age were included to serve as non-AMI controls for RNA sequencing and biochemical analyses.

### ***Cardiac [***^***18***^***F]FDG PET–CMR*** + ***LE***

The acquisition methods and analyses in animal settings were published by our group in detail elsewhere [62]. Briefly, after 12-h fasting of the pigs, serum level of electrolytes and glucose was measured followed by a list-mode ECG-gated PET scan in 3D mode 1 h after intravenous injection of 300 MBq [^18^F]FDG. An intravenous bolus of 0.05 mmol/kg gadobenate dimeglumine (MultiHance®, Bracco Imaging, Italy) was administered for CMR. Time-resolved imaging of three slices in the short axis and one slice in the horizontal long axis was acquired to detect perfusion deficits. Late enhancement (LE) imaging was performed in the short axis and the 4- and 3-chamber views for assessment of the myocardial scar. Cardiac functional parameters, such as left ventricular ejection fraction (EF), end-diastolic volume (EDV), infarct size, MVO [1, 8, 33], and infarct transmurality [45] were calculated as described previously.

Both the CMR and PET imaging were displayed in a 17-segment polar map, and the segmental transmurality by CMR and the [^18^F]FDG normalized tracer uptake [45] were calculated and displayed.

A segment was classified as mismatched if quantified transmurality ≥ 75% paralleled with quantified PET tracer uptake ≥ 75% in the same segment. Animals were assigned to the Mismatch group if at least 3 of the 17 segments were classified as mismatched. The image acquisition was performed by Biograph mMR, Siemens Medical Solutions, Erlangen, Germany.

For the image reconstruction, three-dimensional attenuation-weighted subsets expectation maximization iterative reconstruction algorithm (AW-OSEM 3D) was used [62].

The semiautomatic quantification of the [^18^F]FDG tracer uptake, transmurality, and segmental contractility of the 17-segment model has been described previously [62].

The images were analyzed and supervised by the authors AJ, ZS, SG, who are experienced in radiology and imaging.

### Histology, and circulating and tissue inflammatory parameters

HE staining was performed in accordance with the manufacturer’s instruction. Circulating IL-1beta (RAB0276) and IL-6 (RAB0310, both Sigma-Aldrich, St. Louis, USA) were measured for samples taken at baseline, 30 min and 3 days after reperfused AMI.

Quantitative PCR of the infarcted and border myocardial tissues as well as control non-infarcted hearts were performed to quantify the tissue CD68, CD45, and TNF-alpha expression. To this end, RNA was isolated using miRNeasy (Qiagen) in a QiaCube according to the manufacturer’s instructions. RNA quantity was assessed on a Nanodrop 1000 (Thermo Scientific), and 500 ng were used for subsequent cDNA synthesis with the QuantiTect Reverse Transcription Kit (Qiagen). cDNA was diluted 1:25 and used for qPCR quantification with SYBR Green and 0.3 µM forward and reverse primers.

Primer sequences were CD45 fwd, 5'-GCC ACT TCT CCC ACT CAA GG-3'; rev, 5'-AGT GGT GCG AGC AAG TAA GG-3'; CD68 fwd, 5'-TCC CAG TGA CCA AAC CAT CC-3'; rev, 5'-TTG GAA CAG ATG CTC ACG GA-3'; TNFa fwd, 5'-CTG TGC CTC AGC CTC TTC TC-3'; rev, 5'- AAC CTC GAA GTG CAG TAG GC-3'; HPRT fwd, 5'-CCC AGC GTC GTG ATT AGT GA-3'; rev, 5'-ATC TCG AGC AAG CCG TTC AG-3'; ACTB fwd, 5'-TCA ACA CCC CAG CCA TGT AC-3’; rev, 5'-CTC CGG AGT CCA TCA CGA TG-3'.

### Gene expression analysis

For RNA sequencing, strand-specific libraries were prepared from 500 ng total RNA by poly-A enrichment (NEBNext Poly(A) mRNA Magnetic Isolation Module, NEB, Ipswich, MA, USA) and the NEBNext Ultra Directional RNA Library Prep Kit for Illumina (NEB). Libraries were quality controlled on a Fragment Analyzer (Advanced Analytical Technologies, Ames, IA, USA) and quantified by digital droplet PCR (QX100™ Droplet Digital™ PCR System, Bio-Rad, Hercules, CA, USA) and the ddPCR Library Quantification Kit for Illumina (Bio-Rad). Eight to ten libraries were pooled equimolarly for one HiSeq 2500 lane (Illumina, San Diego, CA, USA) and each library was paired-end sequenced. After demultiplexing, raw reads were quality controlled by FastQC and sub-sequentially mapped to the Sus scrofa genome (Sscrofa10.2) by the RNA-Seq Unified Mapper [14]. Finally, mapped reads were counted into the Sus scrofa Ensembl gene model Sscrofa10.2.

RNA sequencing data were further processed with trimmomatic, limma, GOsummaries and ClusterProfiler for quantification of relative gene expressions and illustration of gene ontologies and KEGG and reactome pathways. Ridgeplots were created with ClusterProfiler (GSEA) and ggplot2. Immune cell infiltration into the myocardium was analyzed using the RNA sequencing results with the ImSig algorithm [40].

### Single-nuclei RNA sequencing

#### Tissue homogenization and nuclei isolation from frozen myocardial tissue

Heart tissue samples (100–200 mg) from the left ventricle were excised and cryostored. For nuclei extraction, the tissue was homogenized using a GentleMACS dissociator (Miltenyi Biotec, Germany) applying the program for single-nuclei extraction 1a. The tissue was placed into a homogenization buffer containing 10% Triton X-100, as previously described [31]. Homogenized tissue was filtered through 70 µm and 40 µm Flowmi tip filters (Merck; Germany). Nuclei were pelleted by centrifugation at 500x*g* for 5 min at 4 °C and stored in Lo-bind tubes in storage buffer containing 5% BSA in PBS and 0.2 U/µl Protector RNase inhibitor (Merck; Germany). Trypan blue staining was performed to acquire images of nuclei and inspect their membrane integrity using a fluorescence microscope (Olympus IX83; Japan) processed with Olympus CellSens software (Shinjuku). Nuclei were stained with AO/PI and counted on a LUNA™ automated cell counter (Logos Biosystems, France) to determine concentration prior to loading onto the 10 × Chromium device (10 × Genomics; CA, USA) (Supplementary Material).

### snRNA-seq and data processing

Single-cell RNA-seq libraries were generated using the Chromium Controller and the Next GEM Single Cell 3ʹ Reagent Kit (v3.1, 10 × Genomics) following the manufacturer’s instructions, with the aim of achieving a maximum cell recovery of 10,000 cells. The libraries were sequenced on the Illumina NovaSeq 6000 platform by the Biomedical Sequencing Facility at the CeMM Research Center for Molecular Medicine of the Austrian Academy of Sciences. Raw sequencing data were demultiplexed using bcl2fastq v2.20.0.422. Subsequently, the data were processed using the cellranger count pipeline (v7.0.0, 10 × Genomics) with a custom reference generated via cellranger mkref, to align the sequenced reads to the S. scrofa genome. The custom reference was built using the Sscrofa11.1 assembly and the corresponding general feature format file. The file was filtered by cellranger mkgtf for protein coding biotype. The reads were mapped to both introns and exons. Each cell barcode was assigned a donor identity based on further demultiplexing of hashtag barcodes. To perform demultiplexing and infer the probability that a given hashtag barcode read count originates from background or constitutes positive signal, a Gaussian mixture model was fitted to hashtag barcode counts, as described previously [24].

### QC of snRNA-seq data, clustering, and visualization

The Seurat pipeline was applied for nuclei quality filtering in all our downstream analysis. Nuclei with at least 200 expressed genes were retained and nuclei with more than 20% genes of mitochondrial origin (ND1, ND2, ND3, ND4, ND5, ND6, ND4L, COX1, COX2, COX3, CYTB, ATP6, ATP8) were filtered out. Sct normalization was done with a total of 10 000 nuclei and values were log-transformed. Filtered expression matrix was used for analysis of each sample. Highly variable genes were identified and dimensionality reduction on the variable genes was performed applying principal component analysis. We identified clusters using the Seurat function FindClusters, following visualization of the results applying Uniform Manifold Approximation and Projection (UMAP) [36]. Data from the pig single-cell atlas database [59] and the heart cell atlas [22] were retrieved to identify gene markers expressed in clusters enabling annotation of clusters.

### Enrichment analysis of cellular clusters across myocardial areas in Mismatch group

To elucidate regulated pathways within the distinct cell clusters, differential gene expression analysis was conducted. This involved comparing differentially expressed genes (DEGs) in individual cell clusters between the Mismatch and Match groups. Analysis was facilitated using the FindMarkers function within the Seurat pipeline, employing the MAST testing methodology, which is specifically designed for single-cell RNA sequencing (scRNAseq) data. Genes exhibiting significant regulation (adjusted *p* value < 0.05) were further analyzed for pathway involvement using the PathfindR tool (version 2.3.0) [55], which focuses on active-subnetwork-oriented gene set enrichment analysis. The resulting enriched terms were subject to hierarchical clustering, utilizing kappa statistics to define distances (distance = 1−kappa statistic).

### Statistics

Continuous parameters were assessed by mean ± standard deviation between the groups. Statistical significance was calculated by comparing the groups using ANOVA with Bonferroni post hoc analyses. Correlations between two continuous parameters were analyzed using regression curves. Statistical significance was stated if *p* < 0.05.

## Results

### Study data

**Fig. 2:**
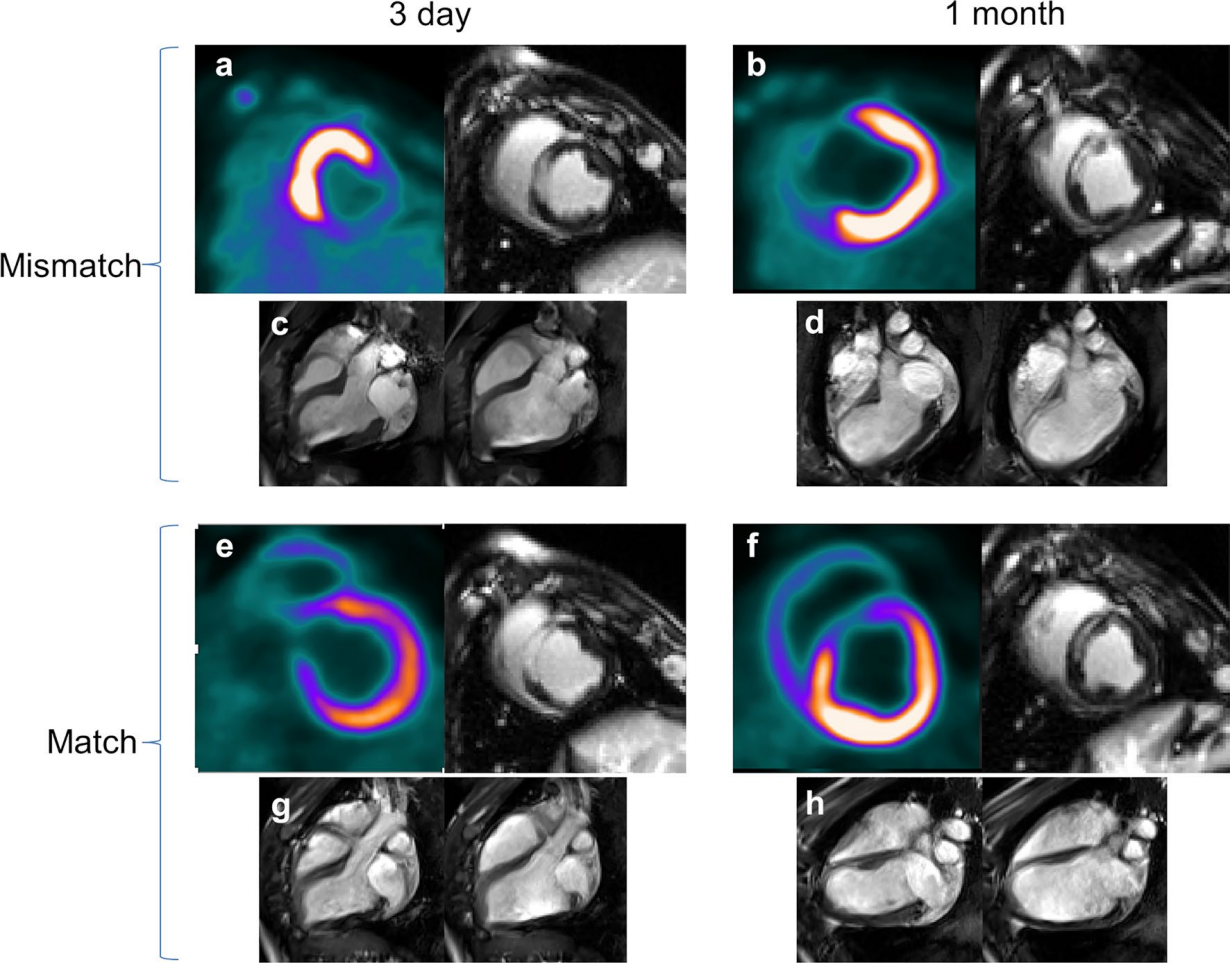
Mismatch and Match between [^18^F]FDG PET and cMRI + LE at 3-day and 1-month post-anterior AMI. **a**. PET/MRI Mismatch at 3 days: high [^18^F]FDG tracer uptake at the site of infarction (left panel), late enhancement at the anteroseptal area corresponding to the infarction by cardiac MRI (right). **b**. PET/MRI Mismatch at 1 month: [^18^F]FDG tracer uptake defect (left) and late enhancement in the infarcted area (right). **c**. MRI at 3 days (Mismatch group): end-systolic (left) and end-diastolic contour (right). **d**. MRI at 1 month (Mismatch group): end-systolic (left) and end-diastolic contour (right), showing large anterior aneurysm and severely reduced cardiac function. **e**. PET/MRI Match at 3 days: [^18^F]FDG tracer uptake defect at the site of infarction (left panel), late enhancement at the anteroseptal area by cardiac MRI (right). **f**. PET/MRI Match at 1 month: [^18^F]FDG tracer uptake defect (left) and late enhancement in the infarcted area (right). **g**. MRI at 3 days (Match group): end-systolic (left) and end-diastolic contour (right). **h**. MRI at 1 month (Match group): end-systolic (left) and end-diastolic contour (right), showing anterior infarction and reduced cardiac function

In our study of percutaneously induced rAMI in pigs (*n* = 30), the blood sugar level was 105 ± 8 mg/dL after overnight fasting, indicating normoglycemic conditions. Coronary angiography 5 min after reperfusion showed TIMI flow 3 in all animals in both groups, except one (8%) in the Mismatch and three (12%) animals in the Match group with TIMI flow 2 (*p* = n.s.); and myocardial blush grade 3 in all animals, except one animal (8%) in the Mismatch and two animals (8%) in the Match group with myocardial blush grade 2 (*p* = n.s.). Coronary angiography before PET/CMR imaging revealed no change in TIMI flow and blush grade in the animals. We detected unusually high [^18^F]FDG uptake in the ischemic myocardial area of eight animals (27%) in routine cardiac combined [^18^F]FDG PET/CMR with LGE imaging performed 3 days after rAMI. This constitutes an apparent mismatch between high tracer uptake and reduced cardiomyocyte viability and contractility at the infarct site displayed by [^18^F]FDG PET and CMR (*n* = 8, group Mismatch), respectively. In contrast, an expected reduced PET tracer uptake was observed in the infarcted area in the remaining pigs (*n* = 22, group Match; Fig. 2). Furthermore, seven additional animals were investigated with PET–CMR at day 3 and allocated to the Mismatch and Match groups (*n* = 3 each, one animal died during AMI), respectively, to allow elaboration of myocardial histology and gene expression patterns that correlate with the viability/metabolism-contraction mismatch. These animals were sacrificed 1 day after the PET investigation to allow decay of the [^18^F]FDG for safety reasons.

### ***Results of combined [***^***18***^***F]FDG PET–CMR***

**Fig. 3:**
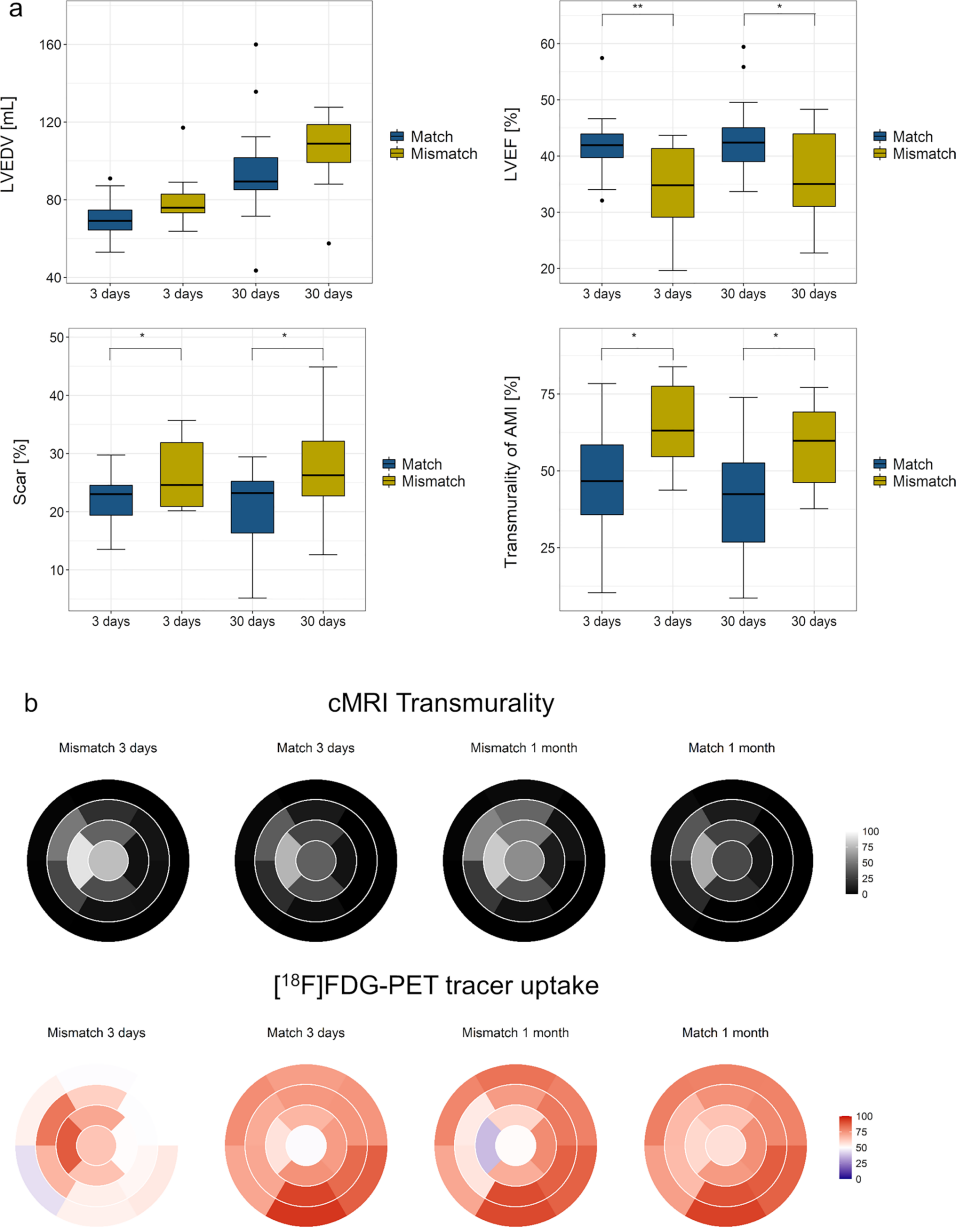
Cardiac magnetic resonance (cMRI) parameters and [^18^F]FDG PET imaging 3 days and 1 month after reperfused myocardial infarction in pigs in groups Match and Mismatch. **a** Quantitative cMRI parameters: left ventricular end-diastolic volume (LVEDV), left ventricular ejection fraction (LVEF), myocardial infarct scar, transmurality of the myocardial infarct scar, *: *p* < 0.05, **:* p* > 0.01. Pigs in the Mismatch group suffered from significantly lower LVEF, had a larger infarct scar, and higher AMI transmurality. **b**. Polar map imaging of the cMRI infarct transmurality (upper panel) and [^18^F]FDG PET tracer uptake (bottom panel). Note the high [^18^F]FDG PET uptake in the infarcted area at 3 days and lower tracer uptake with higher transmurality and larger infarction at 30 days after reperfused myocardial infarction in the Mismatch group

The animals in the Mismatch group had significantly lower EF at 3-day (34.0 ± 8.7 vs 42.0 ± 5.2%, *p* < 0.01) and at the 1-month follow-up (35.8 ± 9.5 vs 43.0 ± 6.3%, *p* < 0.05) as well as a larger infarct area at day three (26.6 ± 6.6 vs 22.1 ± 4.4%, *p* < 0.05) and 1 month (28.0 ± 10.4 vs 20.3 ± 7.2%, *p* < 0.05) with a trend toward higher EDV at 1 month (Fig. 3a). The semi-quantitative normalized segmental [^18^F]FDG uptake of the 17 myocardial segments is displayed in Suppl. Tables 1 and 2. Mean tracer uptake of the infarcted area was significantly reduced in the Mismatch group at 1 month (56.0 ± 23.1 vs 64.7 ± 13.2%). Polar plots of the infarct transmurality by CMR and [^18^F]FDG uptake by PET clearly separated the Mismatch and Match groups (Fig. 3b and Suppl. Fig. S1). In contrast, the 1-month follow-up combined images represented a significant positive linear correlation between [^18^F]FDG uptake and segmental contractility, indicating the resolution of the acute inflammation (Suppl. Fig. S2). Thus, our data in the pig model show that unexpectedly high FDG uptake 3 days after infarction is a prognostic indicator of adverse cardiac remodeling and poor functional recovery.

MVO did not differ significantly between the groups Mismatch and Match (Suppl. Fig. S3). The mean [^18^F]FDG PET tracer uptake was increased significantly in the infarcted segments (apical and mid anterior and apical and mid septal segments) in the Mismatch group. Supposing that MVO is associated with invasion of activated neutrophils releasing neutrophil extracellular traps (NET), we investigated the correlation between the MVO with infarct transmurality and mean [^18^F]FDG PET tracer uptake in the infarcted area. We found no significant correlation between the extent of MVO and [^18^F]FDG PET tracer uptake (Suppl. Fig. S3), indicating that beside NETosis, other cellular mechanisms, e.g., macrophage accumulation, might also play a role.

### Histology of infarction and inflammation biomarkers

**Fig. 4:**
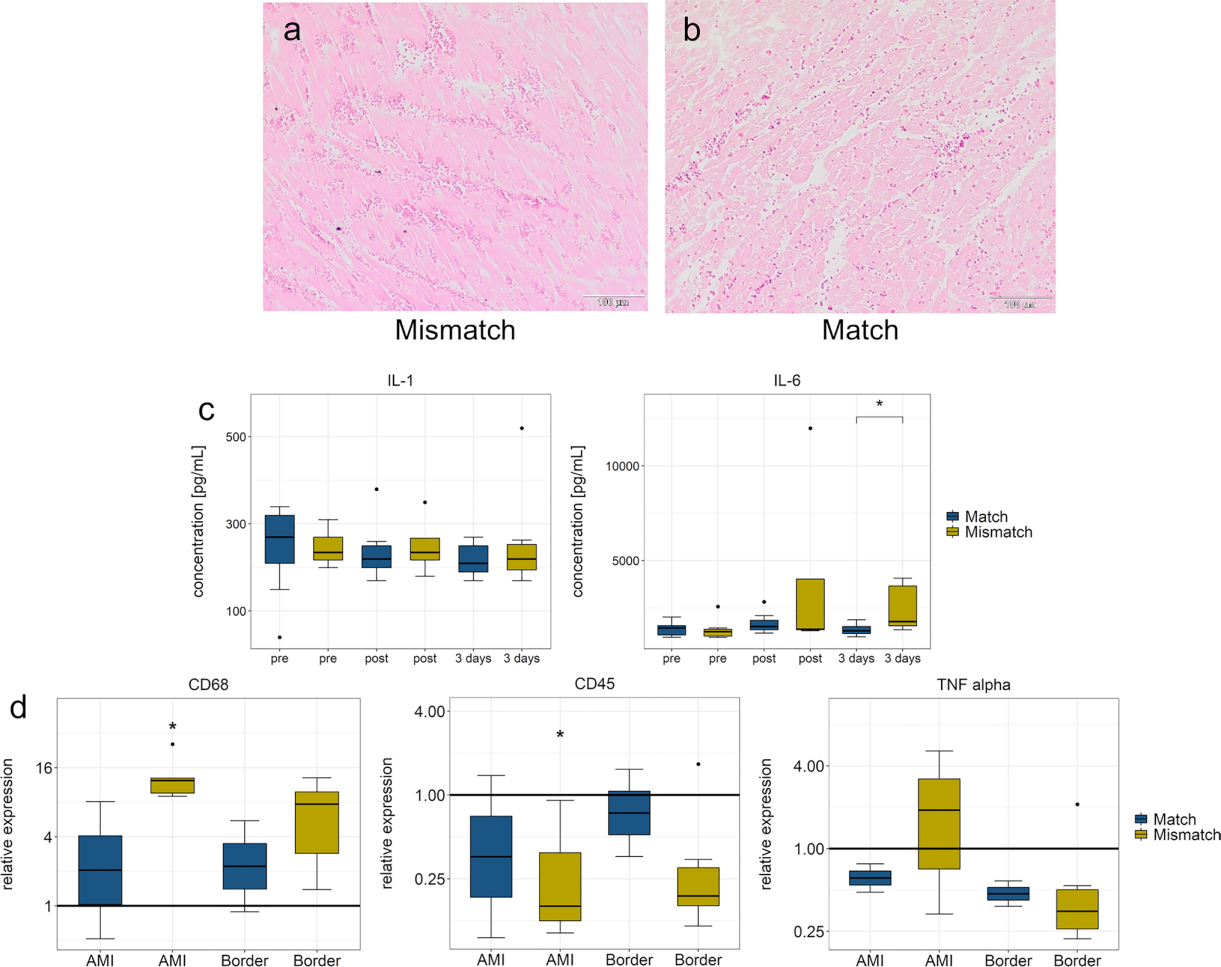
Histology and circulating inflammatory biomarkers in Match and Mismatch groups. **a** and **b** Hematoxylin and eosin (HE) staining of the infarcted areas at day three show haemorrhagia in both Mismatch (**a**) and Match (**b**) groups. **c** Blood biomarkers of inflammation: ELISA of IL-1 and IL-6 at baseline, post-reperfused infarction, and at day three, IL-6 was elevated 3 days after infarction, with a trend toward an increase observed post-AMI, *: *p* < 0.05 compared to control tissue. **d** qPCR of CD68 + , CD45 + and TNF-alpha of the infarcted and border zone tissues at day three. Data are normalized to healthy control myocardium; *: *p* < 0.05 compared to control tissue

Histology analysis displayed massive hemorrhage in the infarcted area in both groups 4 days post-AMI (Fig. 4).

Circulating inflammatory markers of IL-1-beta and IL-6 failed to display significant differences between the groups directly post-AMI, but 3 days later, significantly higher levels of IL-6 in Mismatch group were found (*p* = 0.038; Fig. 4).

The CD68 macrophage marker was significantly elevated in the infarcted area in the Mismatch group at day three post-infarction, compared to normal controls by qPCR (Fig. 4). However, elevation compared to the Match group, was non-significant. Curiously, at the same time point, the expression of common leukocyte marker CD45 was slightly reduced in the Mismatch group. No elevation of TNF-alpha was detected.

### Transcriptomic analysis of coding (messenger) RNAs

**Fig. 5:**
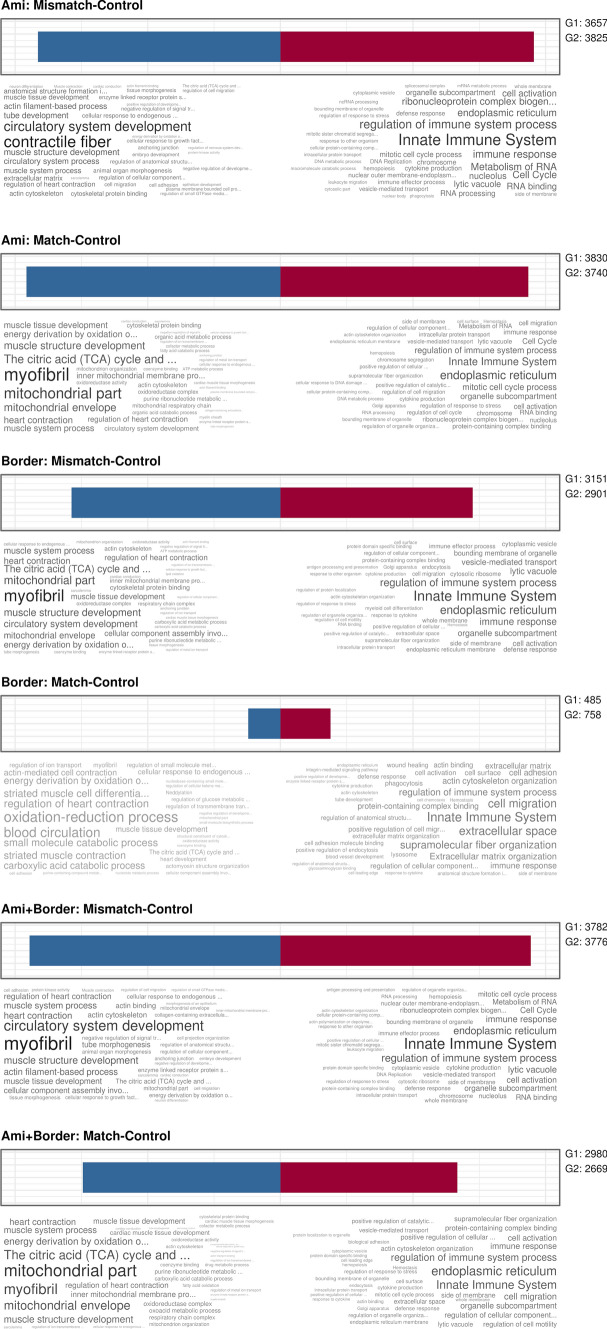
Gene ontology analysis of RNA-seq data. Quantitative data of the transcriptomic datasets (groups Match, Mismatch, and controls) were analyzed using GO summaries. Red and blue bars show the number of downregulated and upregulated genes in the indicated comparisons (e.g., mismatch vs. control in the AMI region). Significantly altered functional groups (GO terms, KEGG, and reactome pathways) are shown in descending order of normalized enrichment size, with shading indicating enrichment *p* values

**Fig. 6:**
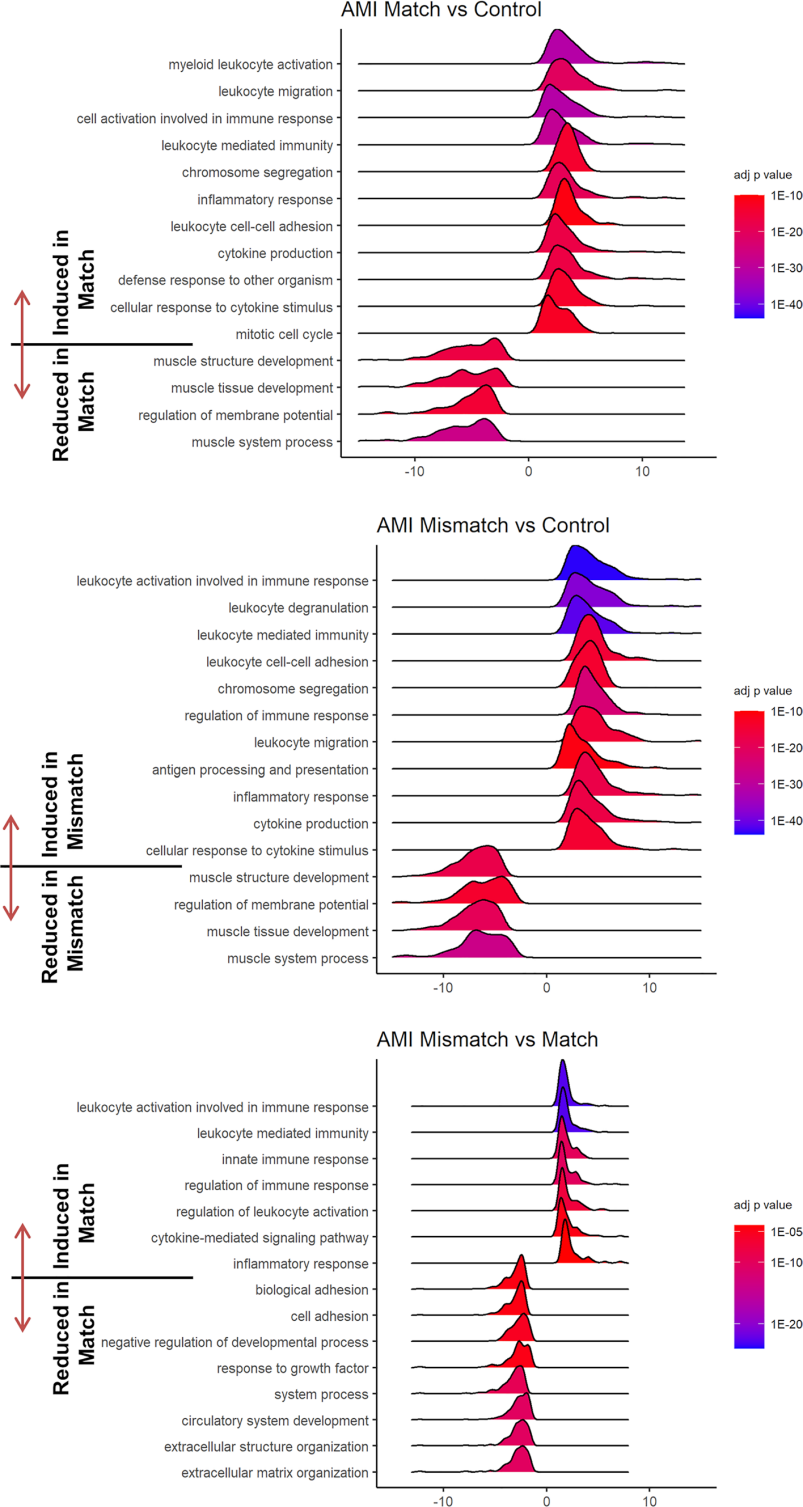
Gene ontology (GO) enrichment analyses of RNA sequencing data. **a** Ridgeplots show the GO terms with the most profound changes between groups. The number of significantly altered genes within each GO term is plotted against the respective log2fold change and fill colors indicate adjusted *p* values. The plots show a uniform change of the listed GO terms, with a predominant induction of immune response genes in infracted tissue of both Match and Mismatch groups compared to healthy myocardium. **b** Direct comparison between the Match and Mismatch group. Stronger induction of leukocyte-related genes and other genes of immune responses in the Mismatch group (positive values indicate stronger gene abundance in Mismatch compared to the Match group). In contrast, gene groups involved in cell adhesion and structural organization were downregulated in the Mismatch group when compared to the Match group. Quantitative RNA sequencing data (log2fold changes between biological groups) were computed by ClusterProfiler using the gseGO function [64]

For elucidation of the molecular mechanism, and whether a viability/infarction location mismatch or an activation of immune cells are responsible, we sacrificed six pigs (three in each of the Mismatch and Match groups) 3 days after infarct induction and 1 day after [^18^F]FDG PET–CMR imaging, and compared the transcriptome of the infarct and border zones to myocardial tissue of three healthy control animals. After RNA-seq, significantly altered coding genes were used to compute gene set enrichment analysis (GSEA) in the R package ClusterProfiler [67]. This analysis showed highly significant de-regulation of immune-system-related GO terms (strongly overlapping gene sets) as the most significantly altered cellular processes (Fig. 5). This could be expected for the comparison of infarcted (both Match and Mismatch animals) vs. healthy myocardium, and reflects the well-established initial immune system activation in the subacute phase (Fig. 5 and Suppl. Fig. S4). The transcriptomic analysis of the corresponding samples at this time point showed a prevalent presence of immune cells both in the infarcted tissue (Fig. 6a, b) and the border area of infarction (Suppl. Fig. S5a and S5b), and a reduced contribution of cardiomyocytes to RNA expression.

The analysis and direct comparison between Match and Mismatch groups revealed significant alterations of GO gene sets related to leukocyte and immune system activation, and inflammatory response mechanisms in the Mismatch group (Fig. 6). Thus, the initial immune activation is triggered by infarction in both Match and Mismatch groups, and its extent is more severe in the Mismatch animals. As expected, the transcriptome showed a stronger increase of immune cell signatures in the infarcted tissue compared to the adjacent (border) region (Suppl. Fig. S5).

**Fig. 7:**
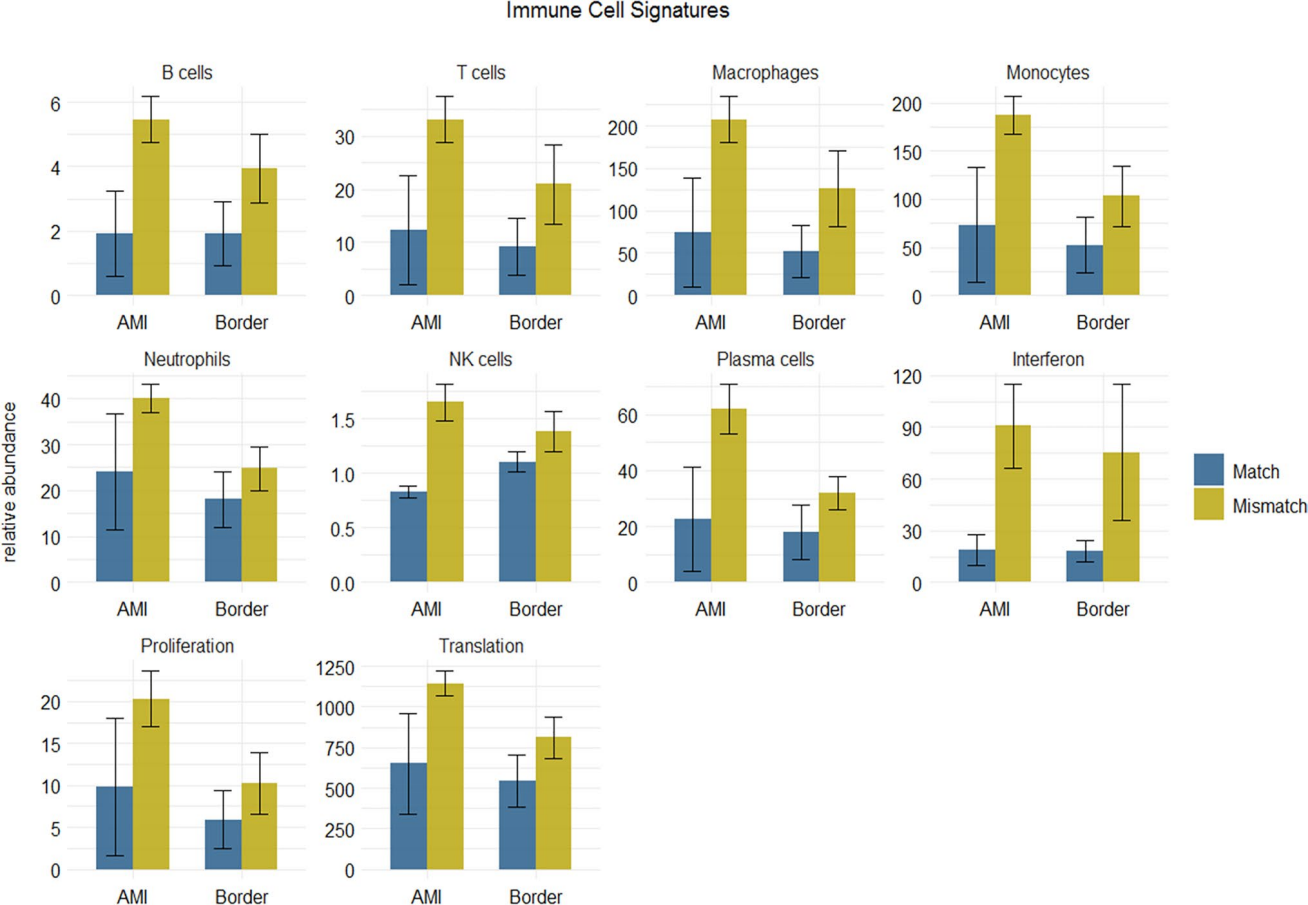
Quantitative analysis of distinct immune cells based on bulk RNA-seq. Using the ImSig algorithm [38], we calculated relative abundances of distinct immune cells in the ischemic region (AMI) and the adjacent region (Border) of pigs with and without mismatching PET and MRI data. We detected high abundance and a strong increase of macrophages and monocytes in the AMI region, and in a lesser extent in the border region of the Mismatch group (not significant). The relative quantities of B and NK cells were also increased in the Mismatch group in the AMI region, but were overall less abundant. These data indicate an overall significantly more intense innate immune response in animals with high FDG uptake, which in turn suffered from a significantly lower EF

A detailed analysis of immune cell abundance based on transcriptomic signatures [40] identified the presence of macrophages and monocytes, but also neutrophils and T cells (Fig. 7). These components of the innate immune response were detected in a higher extent in the ischemic zone, but also in the border region. Our analysis shows that instead of a shift in the immune response (toward different cell types), the immune reaction was overall similar but more intense in the Mismatch group compared to the Match group. The higher abundance of immune cells in pig hearts with higher [^18^F]FDG uptake indicates that the increase in FDG uptake is at least predominantly caused by an excessive infiltration of the transmural infarct by immune cells, and less with a shift from lipid to glucose metabolization, or switch from aerobe to anaerobe metabolization of the subacutely infarcted myocardium.

### snRNA-seq of LV in Match and Mismatch group

To address the limitations associated with bulk-RNA sequencing methodology, single-nucleus RNA sequencing (snRNA-seq) on myocardial tissue samples from both the Match and Mismatch groups was performed. These samples were specifically collected from distinct regions, including the infarcted area, the non-infarcted remote region, and the border region of the left ventricle (LV).

A rigorous quality control assessment was performed (Suppl. Fig. S6), with exclusion of low-quality nuclei exhibiting an abundance of mitochondrial genes (> 20%). The quality of nuclei in the infarcted region with initializing fibrotic mechanism deteriorated due to the necessity for a more intensive dissociation step during nuclei isolation. Consequently, this resulted in a reduction in the number of isolated nuclei, low fraction of reads mapped to transcriptome, and an elevated proportion of mitochondrial genes within the nuclei population (Suppl. Fig. S7).

Subsequently, the Seurat pipeline [17] was employed to integrate datasets originating from each distinct LV region, facilitating a comparative analysis between Match and Mismatch groups. Highly variable genes were identified (Suppl. Fig. S8) and dimensionality reduction on the variable genes was performed to cluster the nuclei (Suppl. Fig. S9). The annotation of clusters was accomplished through a list of conserved markers [50] and further validated using data retrieved from the Heart Cell Atlas database [22].

**Fig. 8:**
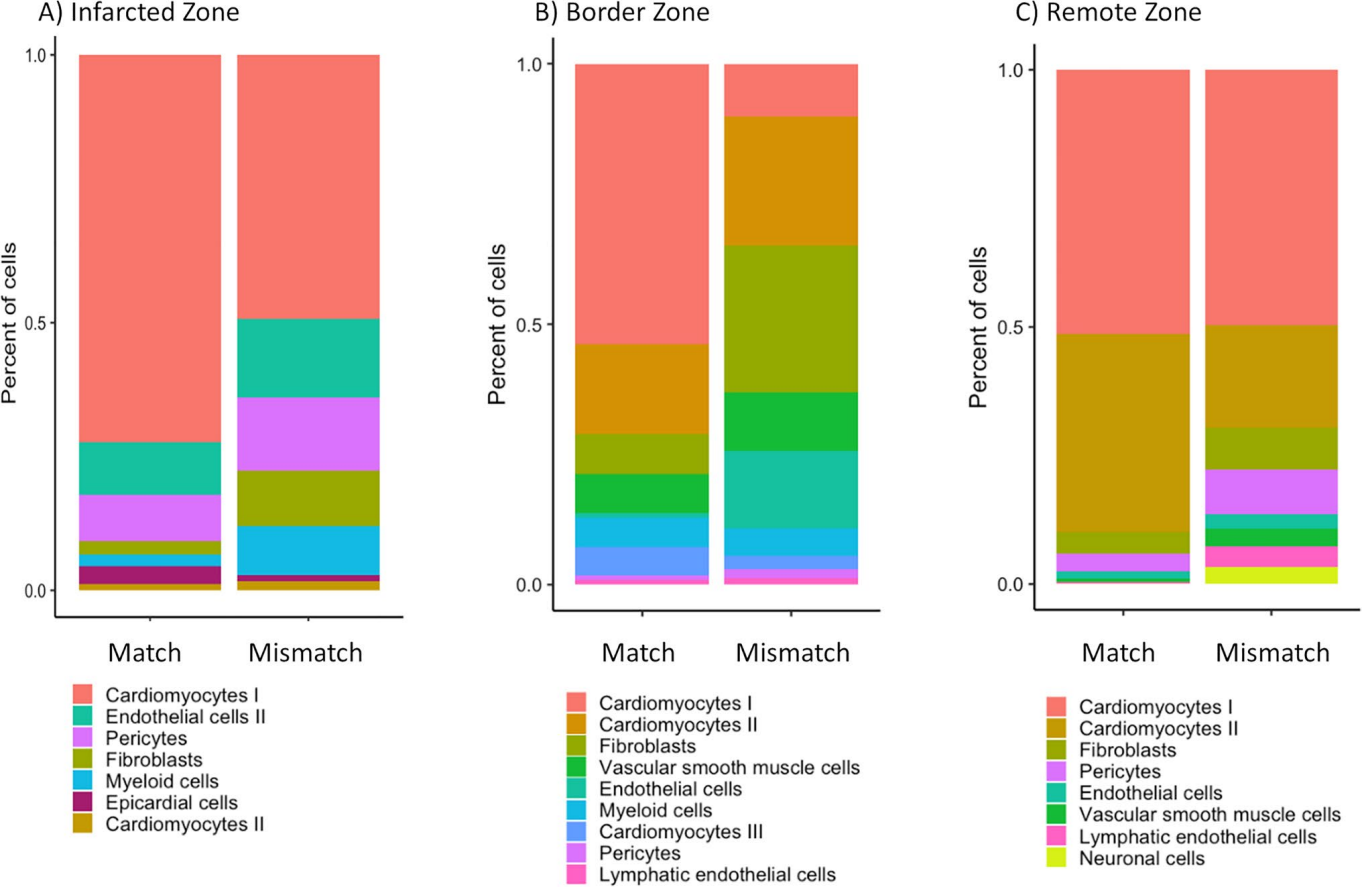
Cell type composition of analyzed nuclei in snRNA-seq. Colors represent cell types labeled according to UMAP in distinct areas of the myocardium (infarct, border, and remote). Higher numbers of non-cardiomyocyte cells were found in the Mismatch group, including lymphocytes in the infarcted zone, myeloid cells in the border zone, and lymphatic endothelial cells in the remote zone

**Fig. 9:**
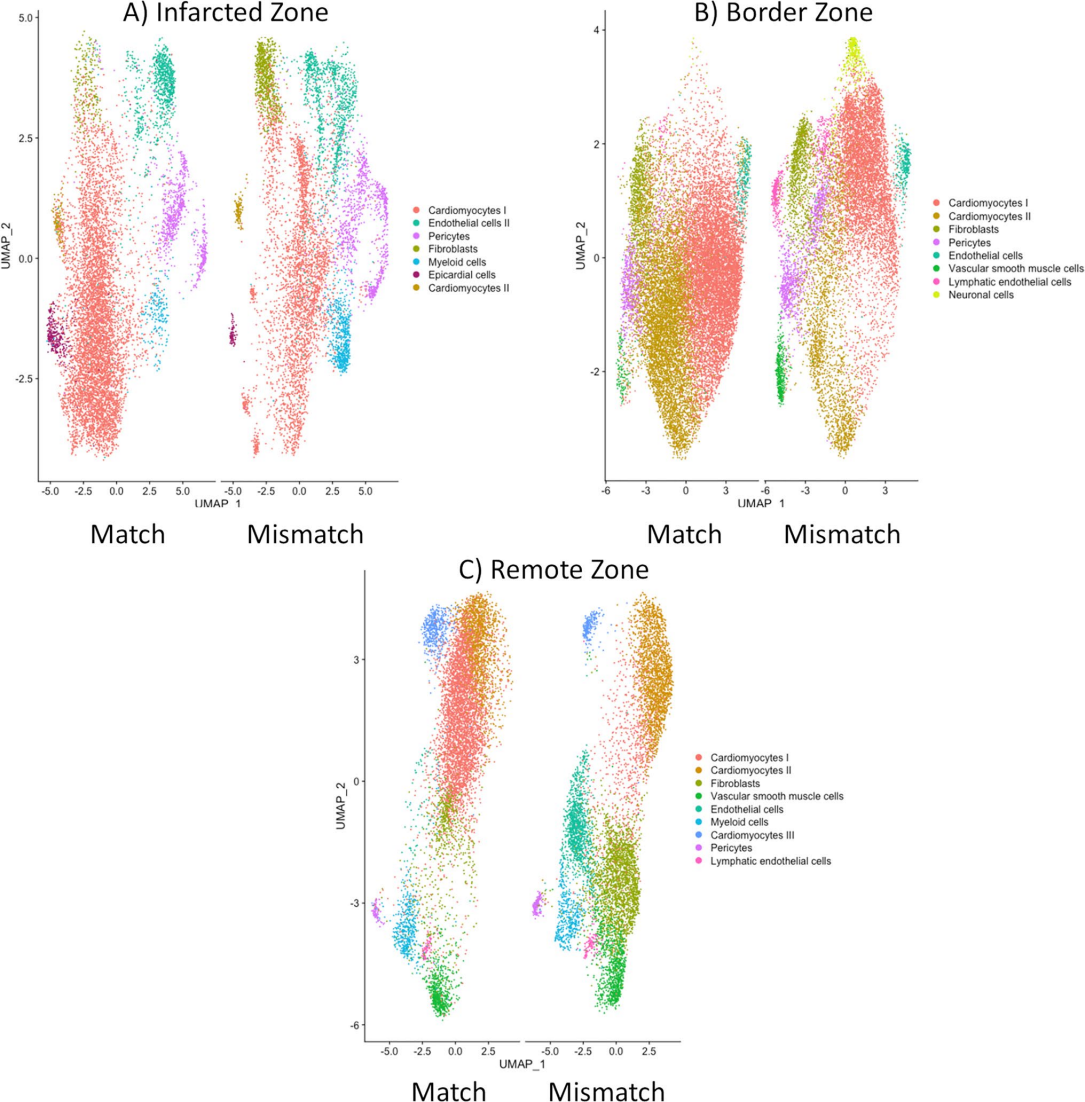
Annotated cell type clusters in the snRNA-seq analysis. Data show integrated datasets of the infarct, border, and remote areas of Match and Mismatch groups. Clusters were identified via the FindClusters function (resolution between 0.4 and 0.8) of Seurat, using principal components with *p* < 0.0001. Data were visualized with the RunUMAP function (reduction = pca)

Confirming previously reported findings [50], the integrated datasets revealed that the most prevalent cell types were cardiomyocytes, followed by fibroblasts, pericytes, endothelial cells, and vascular smooth muscle cells (Fig. 8; Suppl. Table 3). In all three myocardial areas, subpopulations of cardiomyocytes expressing different biomarkers were identified. Cardiomyocytes I exhibited enrichment of TNNI3 and TNNI2, whereas subpopulation II displayed increased expression of ABLIM1, ANKRD1, and MYH7, particularly in the remote and border zones (Fig. 9A, B). A smaller subpopulation, denoted as cardiomyocytes III, was identified, constituting 5% of the Match group and 2% of the Mismatch group in the border zone (Fig. 9B), exhibiting robust expression of RGS6, PDE4D, and PDE3A genes involved in G Protein Coupled Receptor signaling, potentially suggesting a cardioprotective role [46]. Furthermore, small populations of neural cells characterized by the expression of NRXN1 and NKR4 genes, as well as lymphatic endothelial cells enriched for RELN, were identified in both the border and non-infarcted zones (Fig. 9A, B).

These outcomes suggest that the quality of sequenced nuclei has a direct influence on transcriptome data, manifesting in the form of enhanced cluster resolution and more accurate cluster identification. Transcriptome data obtained from the infarcted area (Fig. 9C) revealed the identification of only seven clusters, accompanied by a diminished count of significantly regulated genes in comparison to the border and remote areas. This can be attributed to the lower quality of nuclei extracted from the infarcted area.

### Immune response of cell clusters in border area of the myocardium

An exploratory analysis of differential gene expression was conducted to explore transcriptional variations between the Match and Mismatch groups within individual cell clusters. The data revealed significantly and strongly elevated levels of immune defense activity within several cell clusters of the Mismatch group. Notably, in lymphatic endothelial cells located in the non-infarcted zone, there was an upregulation of genes associated with immune responses, with proportions of 0.5% in the Match group and 2% in the Mismatch group.

A functional GO enrichment analysis showed that immune response mechanisms were upregulated with high significance in all cell types in the border and remote zone. The highest proportion of upregulated genes involved in immune responses was consistently observed in the border zone across all cell clusters within the Mismatch group. Key genes exhibiting significant upregulation in this context included SPP1, IFI6, VIM, RUNX2, CTSS, CTSB, IFIH1, and CD74 (Suppl. Table 4). However, cell populations in the infarcted zone of the Mismatch group did not exhibit any significantly upregulated pathway. This can be attributed to the limited number of upregulated genes identified when comparing the transcriptional profiles between the Match and Mismatch groups (Suppl. Fig. S10).

To investigate potential differences in the immune cell populations between the Match and Mismatch groups, a reference-based annotation approach was employed. To ensure accurate comparisons of cell type markers, data from the Pig Single Cell Transcriptome Atlas [58] were retrieved, serving as a source of annotation markers (Suppl. Table 5).

Although the proportion of immune cells within the population of nuclei was relatively low with less than 5%, the absolute count of distinct immune cell types was notably higher in the Mismatch group within the border zone. We observed a clear distinction in the percentage of nuclei in the Mismatch group of the border zone that expressed markers characteristic of monocytes, NK cells, and M1 and M2 macrophages, in comparison to the Match region (Table 1).

**Table 1:** Representation of particular cell types (absolute counts and percentages) within the LV tissue of Match and Mismatch groups

Cell type	Remote region		Border region		AMI region	
	Match	Mismatch	Match	Mismatch	Match	Mismatch
Memory B cells	25 (< 0.1%)	18 (< 0.1%)	18 (< 0.1%)	811 (1%)	560 (< 0.1%)	1531 (1%)
Naive B cells	165 (< 0.1%)	88 (< 0.1%)	74 (< 0.1%)	3249 (2%)	183 (1%)	1022 (1%)
CD8 + cytotoxic T cells	72 (< 0.1%)	173 (< 0.1%)	7 (< 0.1%)	501 (< 0.1%)	201 (< 0.1%)	173 (< 0.1%)
CD4 + naive T cells	46 (< 0.1%)	44 (< 0.1%)	180 (< 0.1%)	692 (< 0.1%)	679 (< 0.1%)	1727 (1%)
CD8 + naive T cells	27 (< 0.1%)	33 (< 0.1%)	276 (< 0.1%)	2172 (1%)	915 (1%)	695 (< 0.1%)
Monocytes	1247 (1%)	1682 (1%)	1003 (1%)	7643 (5%)	236 (< 0.1%)	688 (< .0.1%)
Neutrophils	26 (< 0.1%)	671 (< 0.1%)	193 (< 0.1%)	622 (< 0.1%)	624 (< 0.1%)	1863 (1%)
NK cells	932 (1%)	1310 (1%)	194 (< 0.1%)	5698 (4%)	253 (< 0.1%)	691 (< 0.1%)
M1 Macrophages	1173 (1%)	1557 (1%)	41 (< 0.1%)	2295 (1%)	288 (< 0.1%)	742 (< 0.1%)
M2 Macrophages	405 (< 0.1%)	754 (< 0.1%)	20 (< 0.1%)	1818 (1%)	318 (< 0.1%)	1464 (1%)

### Pathway enrichment analysis of distinct cell clusters of the Mismatch group

**Fig. 10:**
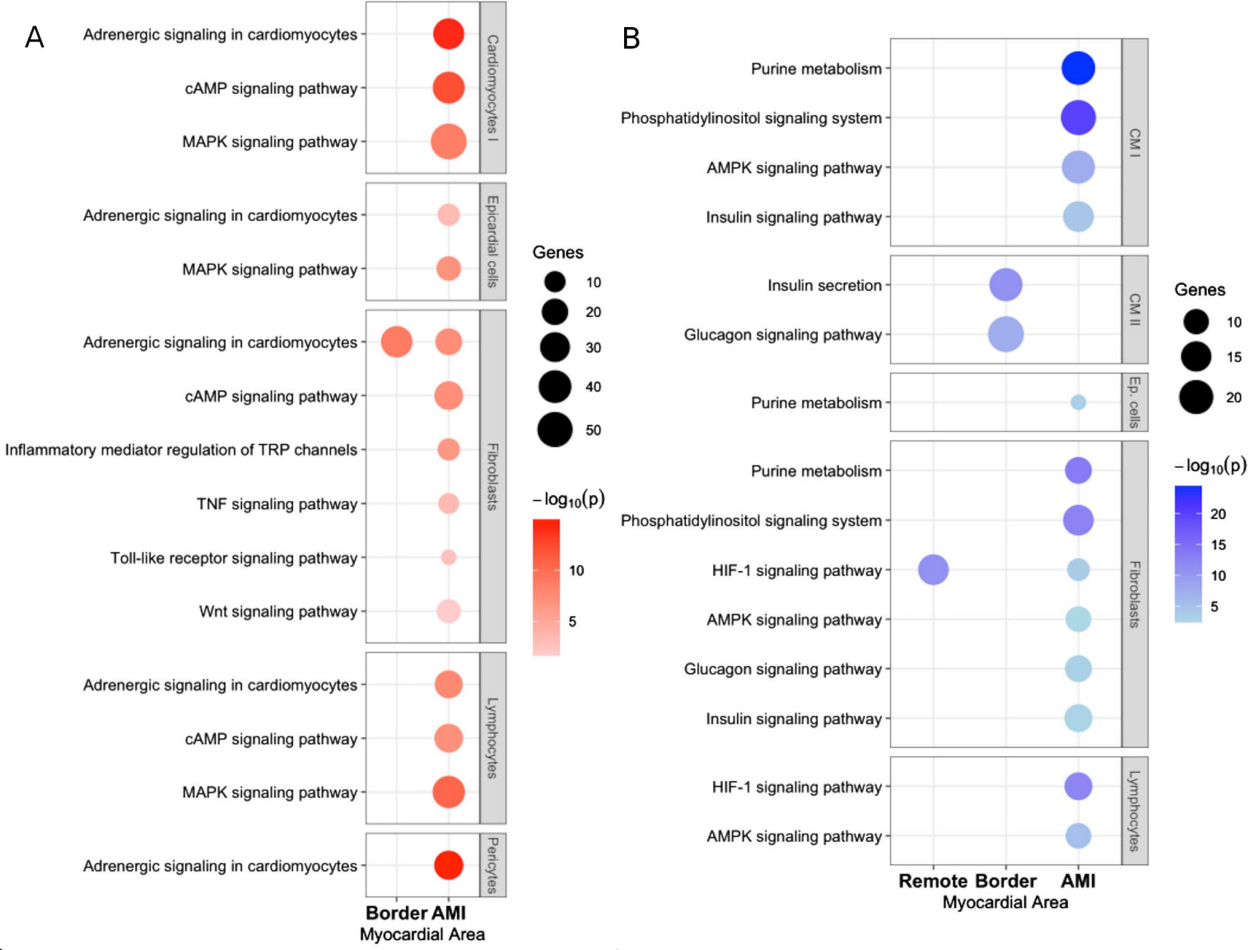
Upregulated molecular pathways in myocardial zones of the Mismatch Group. Bubble charts illustrate the enriched pathways in various cell types from the border and AMI myocardial zones. The size of the bubbles corresponds to the number of genes involved, while the color intensity reflects the significance level (−log10(*p*-value)) of the pathway enrichment. **A** Pro-inflammatory pathways. Adrenergic signaling, cAMP and MAPK pathways were consistently upregulated across distinct cell types in infarcted tissue. **B** Glucose metabolism-associated pathways. The AMPK energy sensor pathway was activated in the AMI zone across cardiomyocytes, fibroblasts, and lymphocytes. Purine metabolism was found to be induced in cardiomyocytes, epicardial cells, and fibroblasts within the AMI area. HIF-1 alpha signaling genes were upregulated specifically in fibroblasts in the remote and AMI region. CM I, cardiomyocytes type I; CM II, cardiomyocytes type II; Ep. cells, epicardial cells

Analysis of snRNA-seq data has delineated distinct cellular profiles, uncovering significant pathway regulation across varied cell clusters (Suppl. Table 6 and Fig. 10A). A pronounced expression of pro-inflammatory molecular pathways within the AMI area of the Mismatch group was observed. Specifically, clusters of cardiomyocytes Type I, pericytes, fibroblasts, lymphocytes, and epicardial cells exhibited upregulation in genes involved in pro-inflammatory pathways (Fig. 10A).

Among the pathways, adrenergic signaling in cardiomyocytes emerged as a particularly prominent pathway. This was evidenced by the upregulated genes coding for the sodium–calcium exchanger (SLC8A1), calcium-voltage-dependent channels (CACNA1C, CACNB2, CACNA2D1, CACNA2D3), and target proteins (RYR2, TNNT2, TNNI3). Such upregulation induced the formation of cAMP, thereby activating the cAMP signaling pathway alongside MAPK signaling (Suppl. Table 6). The impact on fibroblasts in the AMI zone of Mismatch group was profound, with genes involved in Toll-like receptor signaling, TNF signaling, and inflammatory mediator-driven regulation of TRP channels significantly upregulated. Adrenergic signaling observed in cardiomyocytes was similarly activated in fibroblasts located in the border area of the Mismatch group.

In contrast to the infarcted tissue, cell clusters within the remote zone of the Mismatch group did not exhibit any upregulation of genes involved in pro-inflammatory pathways.

The observed mismatch between glucose uptake and myocardial infarction/viability prompted a targeted analysis of glucose metabolism pathways. Consistent with findings related to pro-inflammatory pathways, cell clusters within the AMI area exhibited significant changes. Notably, type I and II cardiomyocytes, epicardial cells, fibroblasts, and lymphocytes demonstrated expression of pathways linked to glucose metabolism (Fig. 10B). Activation of HIF-1 alpha signaling was observed in fibroblasts within both remote and AMI zones, as well as in lymphocytes within the AMI zone, particularly in the Mismatch group. In addition, the AMPK signaling pathway, which serves as an energy sensor, was activated in the AMI zone across cardiomyocytes, fibroblasts, and lymphocytes, leading to the induction of ATP-producing catabolic pathways and glycolysis. This activation potentially influences purine metabolism, which was found to be induced in cardiomyocytes, epicardial cells, and fibroblasts within the AMI area of the Mismatch group. Notably, fibroblasts in the remote zone represented a unique cluster with upregulated HIF-1 alpha signaling genes. These findings suggest a complex network of pathways regulating glucose uptake, which in turn influences myocardial energy metabolism and cellular functions following injury (Suppl. Table 7).

These results indicate that glucose uptake post-myocardial infarction involves a coordinated response from multiple cellular mechanisms during myocardial stress or injury, extending beyond the role of glucose transporters like GLUT1 and GLUT4. We have identified the involvement of Wnt-signaling, cAMP, and MAPK signaling pathways, particularly in the AMI area of the myocardium.

Our findings of the snRNA-seq analysis confirmed an overall much stronger inflammatory reaction and a substantial presence of immune cells in the border area of the Mismatch group, further validating the observations derived from bulk-RNA sequencing.

## Discussion

In this study, we investigated the discrepancy between viability and infarction, as assessed through PET combined with CMR imaging, using a comprehensive suite of molecular biology methods, including bulk-RNA sequencing and single-nuclei RNA sequencing. The converging evidence from these varied techniques enriches our understanding of the molecular dynamics at play in the observed phenomenon.

In pigs, a strong uptake of [^18^F]FDG and a consequent mismatch to contractility assessed in PET–CMR reflects excessive infiltration of immune cells and is prognostic for worse cardiac function. Based on our results, the infiltrating activated inflammatory cells with high metabolic power show increased uptake of [^18^F]FDG compared to severely ischemic myocytes with lower metabolic activity, which results in an apparent display of enhanced metabolism in the area with decreased contractility.

In line with previous reports [60], according to our bulk and snRNA-seq analyses, the most abundant immune cell types 4 days post-infarction were monocytes and macrophages [29], and both were present in a significantly higher abundance in the infarct zone in the Mismatch group. Excessive monocyte infiltration impairs healing and restoration of infarction in mice models [44]. In pigs, inflammatory infiltration of [^19^F]-marked monocytes correlated with LV remodeling and end-diastolic volume [6]. However, macrophage depletion likewise increases ventricular remodeling [56]. A timely and balanced resolution of the initial inflammatory phase is required for limited ischemic damage and subsequent reversed tissue remodeling of AMI [53].

Post-infarct inflammation is a key factor in the outcome of ischemic injury [10]. In the acute inflammatory phase in the first 4 days post-MI, neutrophils, T cells, and pro-inflammatory monocytes and macrophages (M1 macrophages) are recruited to the injured tissue to remove remnants of necrotic cells, and are instrumental in scar formation [46]. In the subsequent proliferative phase, which typically lasts 2 weeks, reparative macrophages (M2 macrophages), and T and B cells modulate cardiac remodeling and activation of fibroblasts. The combined invasion of different inflammatory cells into the subacutely ischemic area might explain that no significant correlation was found between extent of MVO and [^18^F]FDG uptake in the infarcted area, as MVO is associated with NETosis [33]. There is increasing recognition that a balanced immune response is a factor for limiting consequent functional loss, while persisting inflammatory processes can result in excessive tissue remodeling.

Of note, our transcriptomic analyses demonstrated an increase of an innate immune response for both the Match and Mismatch groups compared to healthy myocardium, but the inflammatory reaction was clearly stronger in the Mismatch group, and most pronounced in the border and remote zone. Thus, an excessive and apparently imbalanced innate immune response is associated with worse functional outcome in the porcine MI model. Underrepresentation of immune cells in the AMI region (Table 1) can be explained on the one hand by technical performance of snRNA-seq resulting in differences in sequencing depth across the input libraries that affected the downstream analysis (low post-normalization read depth 39.9%). On the other hand, isolation of nuclei from the MI region required more intense dissociation as compared to the border or remote regions. Besides this inflammatory reaction, the pathway analysis of the direct comparison of Match and Mismatch groups also indicated a downregulation of the circulatory system development pathway (GO biological process). This is an indication of a stronger reparative capacity of the porcine myocardium with lower FDG uptake and further corroborates the detrimental outcome of high myocardial FDG uptake in the acute phase post-MI.

Our snRNA-seq analysis revealed the upregulation of pro-inflammatory pathways specifically in the AMI area of the Mismatch group with prominent upregulation of genes involved in adrenergic signaling pathways. This molecular alternation led to activation of cAMP and MAPK signaling, prominently observed in cardiomyocytes type I, fibroblasts, epicardial cells, lymphocytes, and pericytes. This finding is in line with the research indicating that enhanced adrenergic signaling pathway activates array of chemokines leading to recruitment and infiltration of macrophages within the myocardium [66]. Furthermore, our results showed activation of the Wnt-signaling pathway in fibroblasts, in addition to various pro-inflammatory pathways. This observation is supported by previous research demonstrating the pro-inflammatory capacity of Wnt/β-catenin signaling in models of myocardial infarction (MI) in both rats [68] and mice [58]. Moreover, our findings are consistent with literature suggesting the critical roles of both cAMP [52] and MAPK signaling pathways [21] [49] in the orchestration of pro-inflammatory molecular responses.

Through snRNA-seq, we pinpointed genes implicated in metabolic pathways crucial for glucose utilization. Notably, an activated purine metabolism pathway was identified, coupled with enhancements in glucagon and glucose metabolism pathways within the AMI zone of the Mismatch group, particularly in fibroblasts. This discovery sheds light on the metabolism-infarction/contractility mismatch, possibly due to an imbalance between fatty acid oxidation and glucose oxidation. The latter pathway, known for its superior energy efficiency, utilizes a significant amount of adenosine triphosphate (ATP) for energy provision under ischemic conditions [30]. Pathway enrichment analysis delineated the AMI area of the Mismatch group as a primary locus for molecular processes pertinent to the viability/infarct mismatch phenomenon. The shift of the glucose uptake from the severely ischemic and dying myocytes with lower metabolic activity to the highly active inflammatory cells, and the expression of genes related to pro-inflammatory pathways and myocardial energy metabolism by cardiomyocytes, fibroblasts, and lymphocytes underscores the intricate cellular interplay.

In clinical imaging, infarction sites usually exhibit lower [^18^F]FDG uptake [39]. Still, the data derived from our porcine model showing increased [^18^F]FDG uptake at the infarction site early after AMI correlate with available clinical and preclinical data. Rischpler et al. [47] reported strong [^18^F]FDG uptake in 49 fasted patients 5 days post-infarct particularly in segments with transmural infarction. In this study, performed under conditions that were designed to suppress FDG uptake in normal cardiomyocytes, LGE signals correlated with FDG uptake.

In rodents, Lee et al. [28] showed that high FDG uptake in infarcted myocardium upon ketamine/xylazine anesthesia, which suppresses tracer uptake in the remote myocardium, is largely reflecting inflammation. In human patients, the corresponding high PET [^18^F]FDG signals correlated with peak count leukocytes. In addition, the extent of FDG uptake 5 days after MI correlated inversely with functional outcome at 6 months. Of note, they reported a significant influence of the anesthetic agent on [^18^F]FDG signals. Specifically, the infarct to remote myocardium signal ratio was 1.56 ± 0.1 with ketamine/xylazine and 0.33 ± 0.1 with isoflurane anesthesia. As we used isoflurane in our study, the high [^18^F]FDG uptake in the infarcted region in animals with subsequent worse cardiac function is likely not attributed to a direct influence of the drug regimen on perfusion conditions. However, we did not collect absolute flow data at the time of imaging and a confounding effect of anesthetic agents on perfusion cannot be ruled out entirely.

In the hyperlipidemic HypoE/SRBI^−/−^ mouse model, [^18^F]FDG PET imaging during isoflurane inhalation marked only the viable zones [18], corresponding to the Match group in our study. This study reported no cases of higher [^18^F]FDG uptake at the infarct size. These data might indicate an influence on [^18^F]FDG uptake patterns by species, infarction model, or other parameters.

Independently from the patient or animal preparing protocols or use of diverse anesthetics in animals, all of these studies explained high [^18^F]FDG tracer uptake in the acutely infarcted area by infiltration of metabolically highly active inflammatory cells. Our study is in line with these findings, and explores the cellular and molecular fingerprints of the higher [^18^F]FDG uptake at the reduced contractility area early after myocardial infarction.

In contrast to a hypothetical mechanism that the high FDG uptake is attributed to the switch of the metabolism from lipids to carbohydrates during severe oxygen depletion, transcriptomic analysis did not reveal an appreciable increase in myocyte glucose metabolism. In line with our findings, Borregaard et al. [5] reported enhanced glucose uptake in activated neutrophils. A time-dependent increased accumulation of neutrophils in the AMI area was also observed in mice [9].

In an experiment with seven mini-pigs undergoing induced infarction, Lautamäki et al. reported increased [^18^F]FDG uptake in 68% of CT-defined infarct segments [27]. They showed acute inflammation in these areas as assessed by ex vivo staining and association with microvascular obstruction (no-flow phenomenon). However, no results on subsequent effects on heart function were reported.

Combination of functional images with morphological information (PET/CT, PET/CMR) may stratify patients at high risk and predicted worse clinical outcomes. Beside displaying viable or non-viable myocardium, several simultaneous acquisition technologies have been developed to reveal coronary vessel morphology estimating plaque burden [64], correlating PET/CMR parameter with peripheral blood markers [25], or [^19^F]-perfluorocarbon-nanoemulsions for visualization and quantification of infiltrating monocytes [6], all showing the usefulness of dual imaging parameters as prognostic markers of rAMI. In a study including 369 patients, higher IL-6 levels 24 h after STEMI were shown to be correlated with larger infarct size and decreased cardiac function 4 months post-AMI [15]. We detected higher IL-6 levels in Mismatch animals 3 days after infarction. Our data, thus, show a higher sensitivity of PET–MRI imaging to detect excessive immune response and to predict worse cardiac outcome in our porcine study.

Likewise, the comprehensive bulk and snRNA-seq analyses is clearly superior for analyzing individual immune cell types than quantification of single biomarkers, exemplified by qPCR-based analysis of CD68 and CD45, and quantification of cytokines IL-1 and IL-6.

## Conclusion

Our results prove that an increased [^18^F]FDG uptake in pigs 3 days after rAMI detected in PET/MRI imaging reflects an excessive acute inflammatory reaction after MI, which is driven by infiltration of mainly monocytes and macrophages, but also other immune cells. Enhanced accumulation of inflammatory cells resulted in high [^18^F]FDG uptake in the infarcted area, mainly due to the glucose consumption by the inflammatory cells. This is associated with subsequent worse reparative capacity and is, thus, a prognostic indicator for worse adverse LV remodeling and function. Importantly, the PET/MRI imaging modality enabled differentiation between a normal and an adverse extent at the stage of the initial inflammatory response. These data show that PET/MRI imaging in pigs is a useful preclinical model for investigating potential treatment options aimed at modulating the early immune response. PET/MRI imaging may have prognostic value for early stratification of patients post-AMI. Further preclinical investigations are required to fully evaluate the translational value and to dissect the causative role of inflammation for adverse cardiac remodeling and for devising strategies for more efficient MI treatment.

### Clinical perspectives

Early reperfusion strategy is of utmost importance to salvage the myocardium [19]. The early pathological processes at cellular and subcellular levels determine the infarct size with its high prognostic value. Our translational study on rAMI revealed the prognostic value of the early and massive invasion of inflammatory and other immune-regulatory cells into the ischemic injured myocardium with de-regulation of many genes, revealing several diagnostic and prognostic markers. Based on our results, the non-visible cellular and molecular events can be displayed macroscopically by combined PET/CMR images at the patient level early after myocardial infarction. The here described metabolism-contractility mismatch is associated with worse cardiac function at the follow-up, and is of potential prognostic value in a clinical point of view.

### Limitation

Our study has several limitations. First, various clinical protocols are used to diagnose different cardiac diseases using imaging technologies [43]. High-fat, low-carbohydrate diet followed by a 12-h fasting protocol is predominantly used for imaging of inflammation, such as cardiac sarcoidosis, or vasculitis, while high glucose load with insulin administration with/without heparin prior to myocardial [^18^F]FDG PET imaging is useful to investigate myocardial tissue viability. Both fasting and hyperglycemia have been shown to reduce the myocyte uptake of [^18^F]FDG [4, 42]. Similar to other animal studies with myocardial [^18^F]FDG PET, we have not used dietary restrictions or insulin administration to avoid the metabolic imbalance in the anesthetized animals undergoing PET/MRI investigation [2, 34, 52, 63, 65]. Second, indeed, various anesthetic agents are used as premedication or during porcine [^18^F]FDG PET investigations (telazol, xylazine, isoflurane, propofol, diazepam, and fentanyl) [2, 34, 52, 63, 65]. Anesthesia and anesthetic agents influence [^18^F]FDG uptake in various tissues in anesthetized animals. In contrast to ketamine, isoflurane elevated the blood glucose level only mildly in mice and increased the uptake in the myocardium [11]. As we have used the same protocol in all animals, including feeding, housing, medical treatment, angiography, anesthesia, and PET/CMR images, it seems improbable that different methodologies are causing the variations in [^18^F]FDG patterns in the Mismatch and Match groups, but additional studies with high statistical power might be required for excluding all of these confounders.

To minimize the acquisition time of the anesthetized animals with myocardial infarction, we have not performed perfusion imaging at this time point. However, we have calculated the normalized [^18^F]FDG tracer uptake of the myocardium divided into 17 segments, which has shown the tracer uptake distribution in the entire myocardium. We have included the semi-quantitative normalized [^18^F]FDG uptake values in Suppl. Table 1. In addition, we have previously shown a significant negative correlation between segmental [^18^F]FDG uptake and infarct transmurality only 1 month after myocardial infarction in pigs, but not at day three, presuming also an early invasion of the inflammatory cells into the severely ischemic myocardium [62], suggesting [^18^F]FDG as an inflammatory marker early after ischemia onset and viability marker in the chronic phase of AMI.

The myocardial sampling for RNA-seq and snRNA-seq was performed 1 day after the PET/CMR imaging, due to radiation safety reasons; therefore, the molecular and cellular processes have a 1-day delay to the images. To keep the 3R principles, we have sacrificed three animals per group for the RNA-seq analyses, with a possible negative impact on the statistical power. However, quality control analysis of the samples revealed high homogeneity of gene expression and cellular content within the groups.

Bulk and single-nucleic RNA sequencing have inherent limitations. These technologies offer indications, but not direct proof of involved cell types. The non-linear UMAP clustering method annotated general categories of immune cells such as lymphocytes and myeloid cells. Specific immune cell subtypes have been quantified using a marker-based approach. The correlation between mRNA expression data and protein function can be ambiguous. Pharmacologic inhibition or transgenic knock-out animal models are required for clearer mechanistic proof, but were out of the scope for this study.

CMR became the gold standard of evaluation of myocardial function and infarct size after AMI. Beyond its undisputable value in diagnostics and prognostication of long-term outcome of ischemic heart disease, this imaging modality has also several limitations, such as lack of prognostic viability or local perfusion information in the ischemic area. Combined perfusion/metabolism/functional imaging such as PET/CMR allows fine-tuning of the prognostic information for long-term clinical outcome.

Beyond usual CMR indices (LGE, MVO, infarct size, etc.), further parameters, such as global longitudinal strain, peak ejection and peak filling rates, early systolic lengthening, post-systolic shortening and atrial remodeling are important additional parameters for assessment of the systole-diastolic cardiac function by CMR. However, several of these parameters are not yet validated, especially not for translational MI, and do not give information on cardiac metabolism. In addition, the analysis of further CMR parameter was outside of our study aims, as we focused on the cellular and molecular changes.

## Supplementary Information

Below is the link to the electronic supplementary material.

Supplementary file1 (PDF 2121 KB)

Supplementary file2 (XLSX 13 KB)

Supplementary file3 (XLSX 30 KB)

Supplementary file4 (XLSX 13 KB)

Supplementary file5 (XLSX 13 KB)

Supplementary file6 (XLSX 11 KB)

## Data Availability

Data are available upon reasonable questions.
