# Supplementary material for: Increased [18F]FDG uptake in the infarcted myocardial area displayed by combined PET/CMR correlates with snRNA-seq-detected inflammatory cell invasion
Source: Basic Res Cardiol. 2024 Jun 26;119(5):807–29. doi: 10.1007/s00395-024-01064-y (PMC11461641; doi:10.1007/s00395-024-01064-y)
Supplement: Supplementary file 1 — Supplementary file1 (PDF 2121 KB) [file 395_2024_1064_MOESM1_ESM.pdf]

Supplementary Material

Increased [^{18}F]FDG uptake in the infarcted myocardial area displayed by combined PET/CMR correlates with snRNA-seq-detected inflammatory cell invasion

Dominika Lukovic, Mariann Gyöngyösi, Imre J Pavo, Julia Mester-Tonczar, Patrick Einzinger, Katrin Zlabinger, Nina Kastner, Andreas Spannbauer, Denise Traxler, Noemi Pavo, Georg Goliasch, Dietmar Pils, Andras Jakab, Zsuzsanna Szankai, Ina Michel-Behnke, Lu Zhang, Yvan Devaux, Senta Graf, Dietrich Beitzke, Johannes Winkler*

Supplementary Table 1: Semiquantitative normalized [^{18}F]-FDG tracer uptake in the 17 myocardial segments

**18F-FDG tracer uptake (semiquantitative normalized)**	**Mismatch group 3 days follow-up (n=8)**	**Match group 3 days follow-up (n=22)**	**Mismatch group 30 days follow-up (n=8)**	**Match group 30 days follow-up (n=22)**
Basal anterior [%]	49.5 ±16.7	72.8 ±11.4	82.6 ±15.5	75.5 ±11.0
Basal anteroseptal [%]	53.3 ±22.4	76.8 ±14.9	79.9 ±21.6	77.4 ±10.2
Basal inferoseptal [%]	44.1 ±17.6	71.0 ±12.8	70.7 ±14.7	69.4 ±9.5
Basal inferior [%]	53.3 ±24.7	94.8 ±7.8	91.5 ±7.9	89.5 ±12.0
Basal inferolateral [%]	55.1 ±26.7	87.1 ±11.2	86.5 ±7.9	85.6 ±11.8
Basal anterolateral [%]	49.9 ±22.0	74.9 ±8.6	79.5 ±6.2	78.1 ±10.6
Mid anterior [%]	61.1 ±22.4	74.3 ±14.2	73.6 ±18.0	74.8 ±12.4
Mid anteroseptal [%]	81.8 ±16.1	69.8 ±16.8	54.8 ±21.7	66.4 ±13.2
Mid inferoseptal [%]	67.5 ±15.8	70.6 ±13.2	56.3 ±15.6	65.4 ±13.9
Mid inferior [%]	53.8 ±21.6	92.5 ±8.1	90.9 ±8.7	88.9 ±11.5
Mid inferolateral [%]	52.0 ±24.4	84.6 ±7.6	86.7 ±8.3	86.6 ±11.4
Mid anterolateral [%]	49.6 ±21.4	75.0 ±12.8	81.3 ±5.0	76.8 ±8.2
Apical anterior [%]	70.7 ±21.1	67.3 ±15.1	59.6 ±17.0	63.4 ±12.5
Apical septal [%]	87.9 ±26.5	56.7 ±20.4	37.2 ±14.0	58.3 ±20.6
Apical inferior [%]	64.4 ±20.0	75.7 ±9.8	69.3 ±13.1	73.0 ±10.7
Apical lateral [%]	50.6 ±21.7	73.3 ±8.3	77.1 ±8.8	75.2 ±12.0
Apex [%]	63.9 ±20.7	49.2 ±12.7	50.9 ±14.3	59.4 ±11.5

Red color: $p<0.05$ between the Match vs Mismatch groups at 3 days follow-up
Blue color: $p<0.05$ between the Match vs Mismatch groups at 30 days follow-up

Supplementary Table S2. Segmental contractility data

	Mismatch group	Match 3d	Mismatch 30d	Match 30d
**Segmental contractility**	(n=8)	(n=22)	(n=8)	(n=22)
Basal anterior [cm/s]	21.0±1.7	21.0±1.6	21.6±1.1	23.7±1.5
Basal anteroseptal [cm/s]	22.1±1.6	22-1±1.4	22.3±1.7	23.8±2.3
Basal inferoseptal [cm/s]	21.5±1.3	21.8±1.6	21.2±1.3	23.6±2.0
Basal inferior [cm/s]	21.4±1.6	22.1±1.0	22.2±1.0	23.9±1.3
Basal inferolateral [cm/s]	21.7±1.5	21.8±1.4	23.1±1.0	24.1±1.9
Basal anterolateral [cm/s]	21.2±1.5	21.5±1.6	22.4±0.8	21.8±2.4
Mid anterior [cm/s]	20.9±2.2	21.1±1.7	20.6±1.6	22.0±1.7
Mid anteroseptal [cm/s]	20.0±1.5	20.8±2.3	21.0±1.9	22.1±2.3
Mid inferoseptal [cm/s]	20.5±1.2	20.8±1.6	19.3±2.2	21.6±2.2
Mid inferior [cm/s]	19.4±2.2	21.4±1.7	23.1±1.0	23.0±1.8
Mid inferolateral [cm/s]	20.7±1.5	21.1±1.6	22.1±1.3	23.0±1.5
Mid anterolateral [cm/s]	20.3±1.3	20.4±1.7	20.9±1.9	21.8±1.6
Apical anterior [cm/s]	18.4±2.1	18.3±2.5	18.8±2.2	20.1±2.3
Apical septal [cm/s]	17.4±1.6	18.4±2.0	17.2±1.8	20.2±2.4
Apical inferior [cm/s]	18.7±1.4	19.9±1.8	19.5±1.4	21.1±1.3
Apical lateral [cm/s]	19.1±0.9	19.6±1.5	19.8±1.4	21.5±1.7
Apex [cm/s]	16.2±0.8	18.0±2.7	16.8±1.8	18.4±1.7

Blue color: $p<0.005$ between Match and Mismatch groups

Supplementary Figures

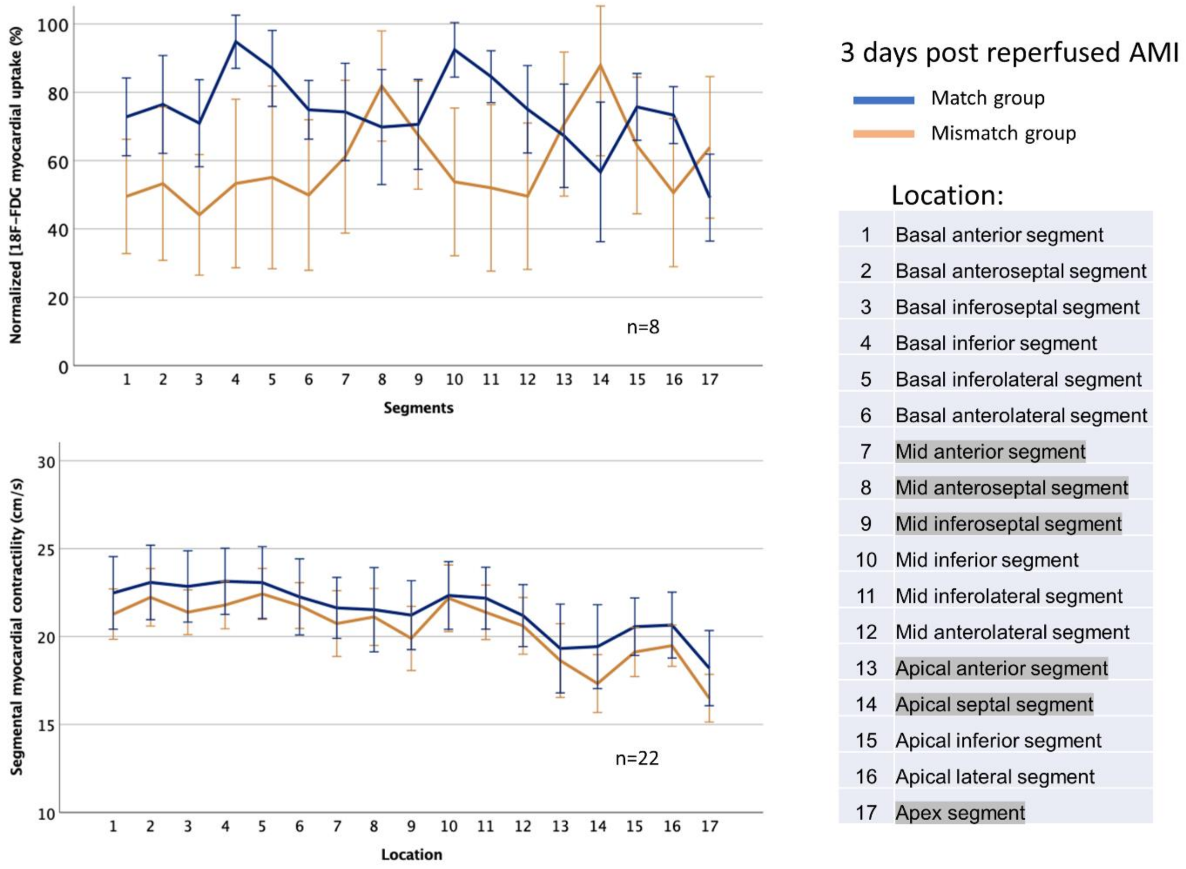

Supplementary Fig. S1. Segmental myocardial [^{18}F]-FDG tracer uptake and segmental contractility 3 days after reperfused myocardial infarction.

Upper panel: Decreased tracer uptake in the infarcted area (segments 7-9, 13,14, 17) in the Match group (blue line). In contrast, relatively increased tracer uptake in the infarcted areas and lower tracer uptake in the remote non-ischemic areas in the Mismatch group (orange line).

Bottom panel: Decreased segmental contractility in infarcted segments in both groups.

Grey segmental areas represent infarcted areas.

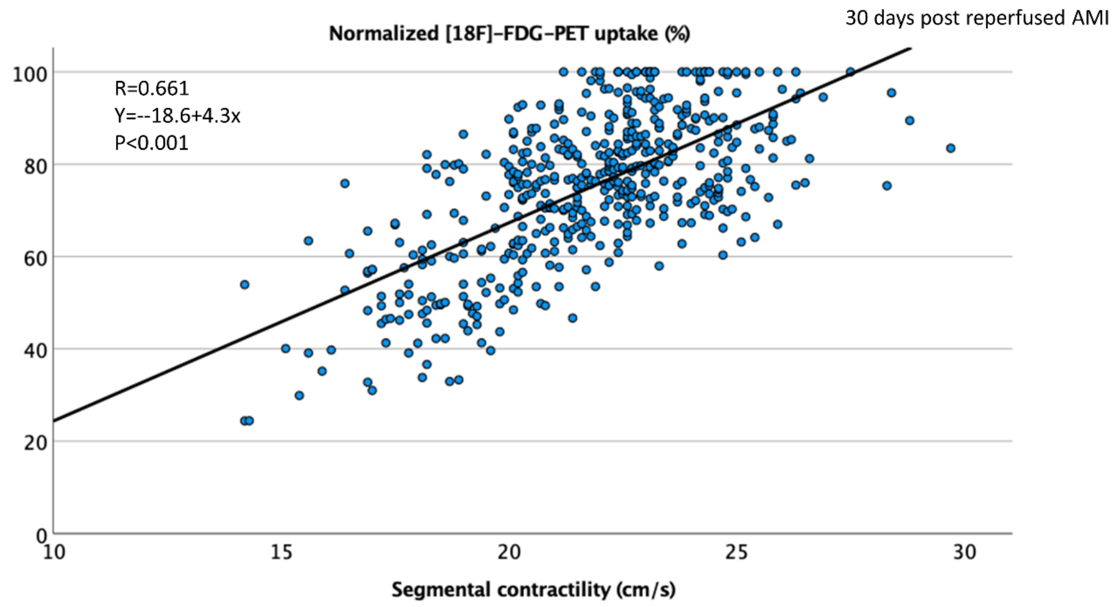

Supplementary Figure S2. Significant correlation between segmental contractility and [^{18}F]-FDG tracer uptake 1 months after the infarction.
Pooled data of all animals.

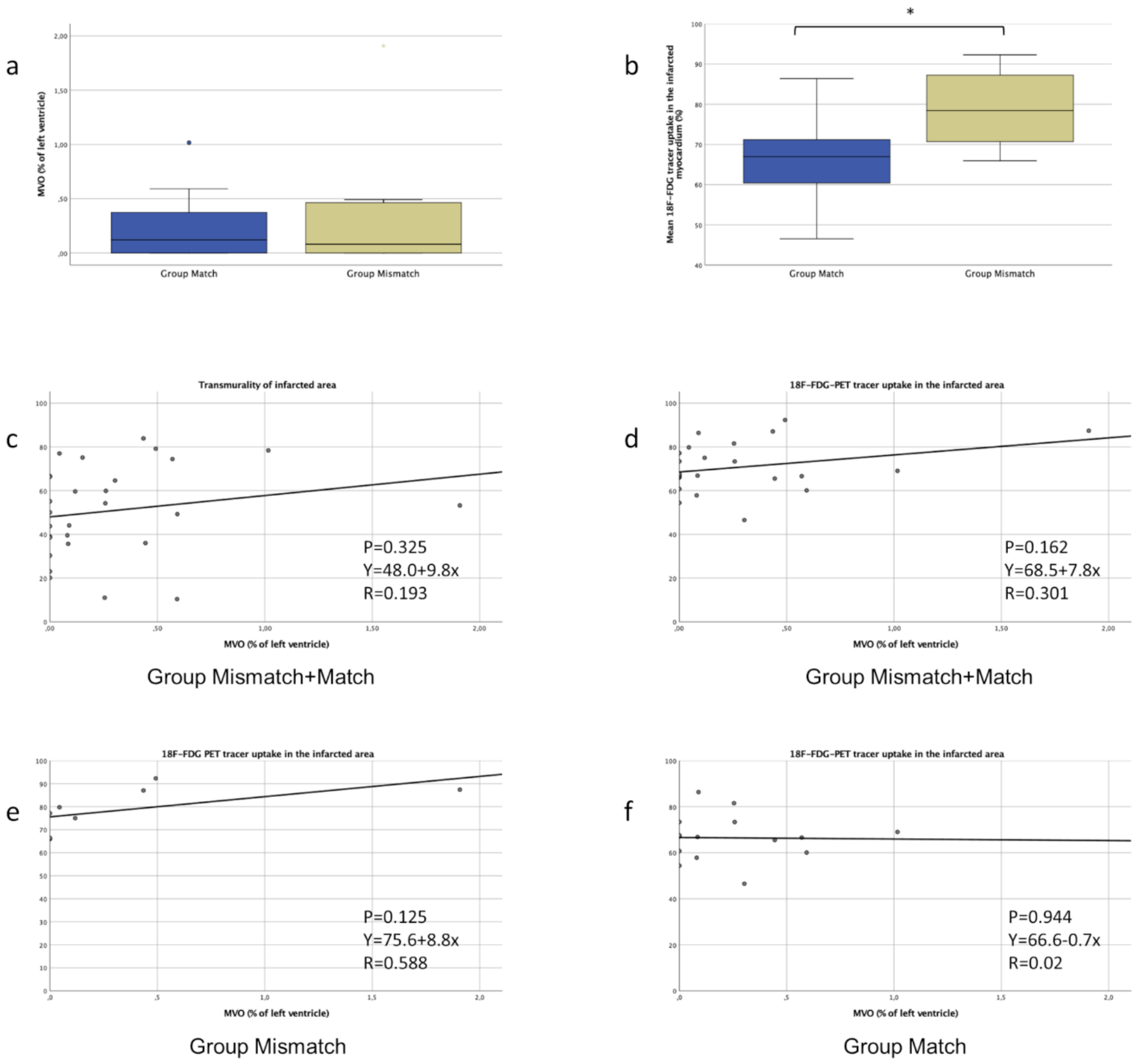

Supplementary Fig S3. Effect of microvascular obstruction (MVO) on infarct transmurality and [^{18}F]-FDG PET tracer uptake in the infarcted area 3 days after reperfused AMI

*a: Quantitative assessment of MVO (percent of left ventricle), b: Quantitative assessment of [^{18}F]-FDG tracer uptake in the infarcted area *:$p<0.05$, c: Insignificant correlation between infarcted area transmurality and MVO in pooled groups, d: Insignificant correlation between [^{18}F]-FDG tracer uptake in the infarcted area and MVO in pooled groups, e: Insignificant correlation between [^{18}F]-FDG tracer uptake in the infarcted area and MVO in Mismatch group, f: Insignificant correlation between [^{18}F]-FDG tracer uptake in the infarcted area and MVO in Match group*

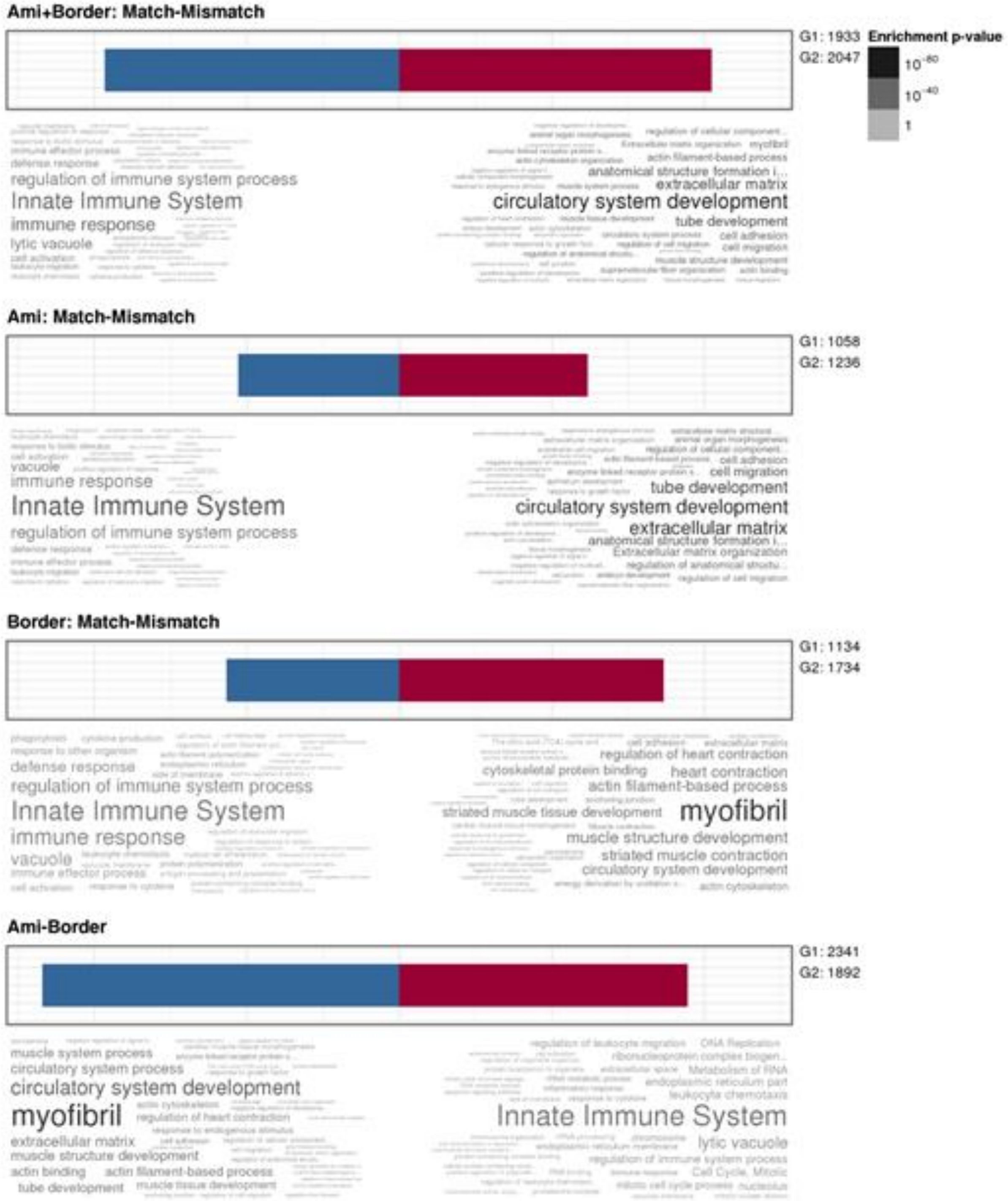

***Supplementary Figure S4. Direct comparison of infarct with (mismatch) and without (match) high FDG uptake** show a strong correlation with activation of the innate immune system in the mismatch group. Genes associated with circulatory system development were downregulated in the mismatch group compared to the match group. Red and blue bars indicate number of up- and downregulated genes. The size and grey scale of gene sets show the statistical significance of the regulation of the respective cellular process.*

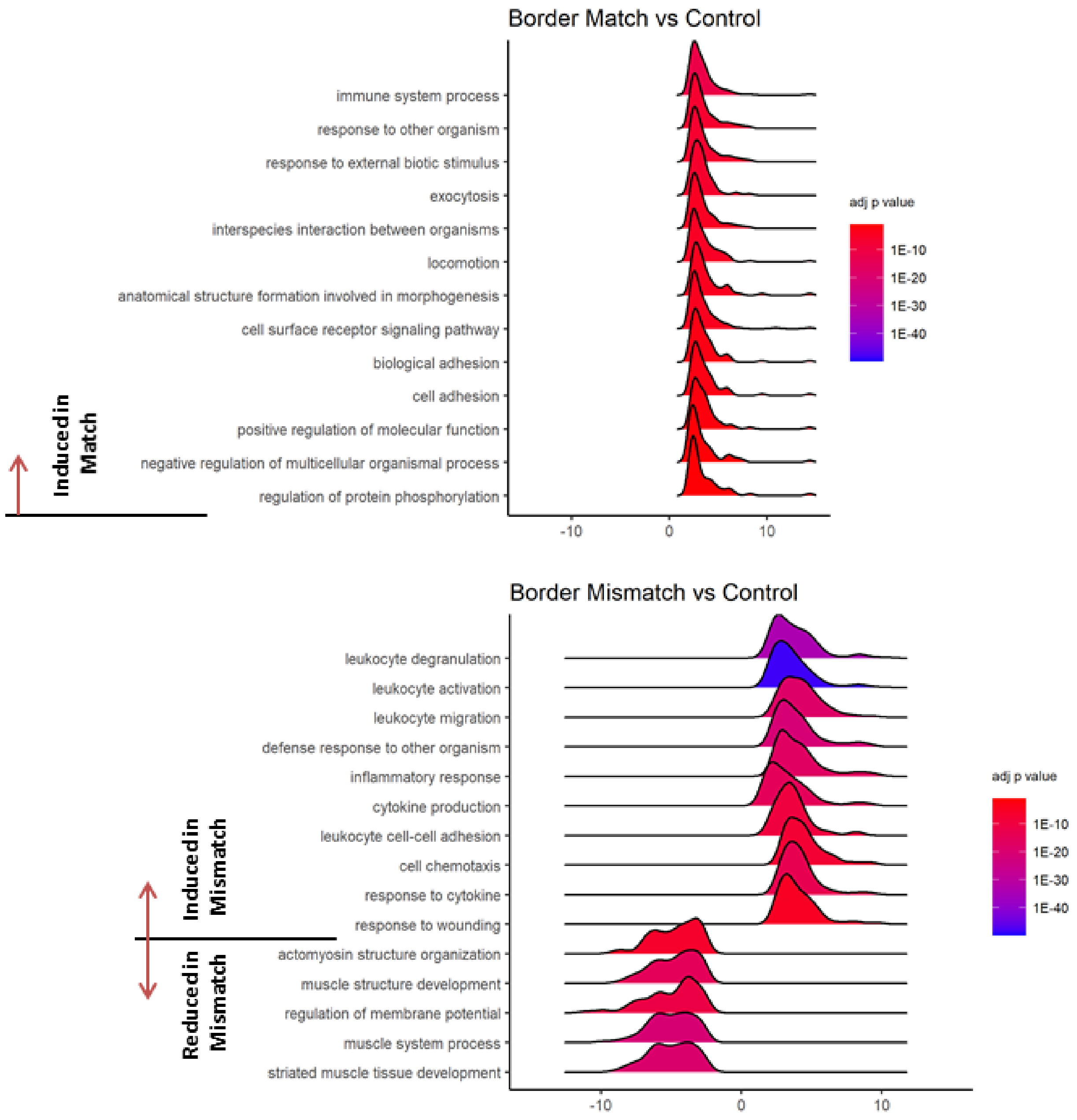

Supplementary Figure S5a. Gene ontology (GO) enrichment analysis of RNA sequencing data in the border zone. *The ridgeplots show the GO terms with the most profound changes between groups. The number of significantly altered genes within each GO term is plotted against the respective log2fold change and fill colors indicate adjusted p values. In the border zone, gene clusters of immune system regulation, particularly genes of leukocyte regulation, were stronger affected in Mismatch animals. Quantitative RNA sequencing data (log2fold changes between biological groups) were computed by ClusterProfiler using the gseGO function.*

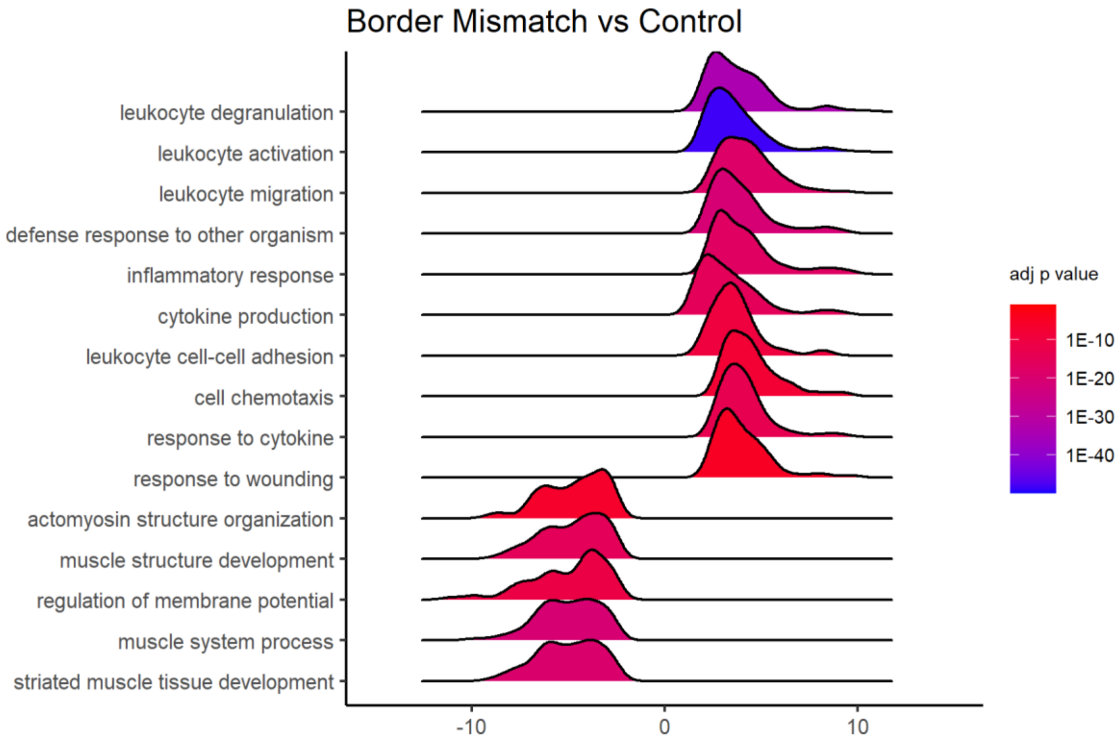

Supplementary Figure S5b. Direct comparison of gene expression between the Match and Mismatch groups in the border zone of the infarcted myocardial area. *The ridgeplots show the GO terms with the most profound changes between groups. A comparison of the border zones between Mismatch and Match animals shows relatively minor differences. Gene sets of muscle system and structure development were significantly stronger expressed in the Match group. Negative values indicate stronger gene abundance in Match compared to the Mismatch group,*

Match Infarcted zone

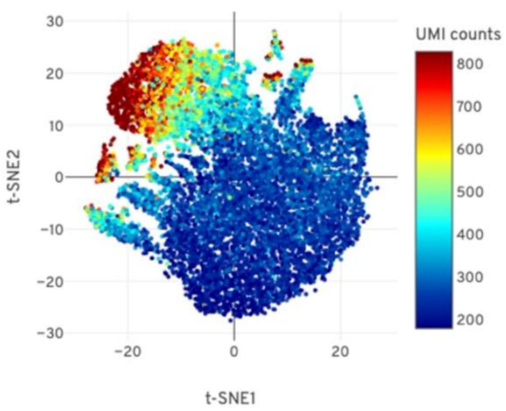

Estimated Number of Cells	76 209
Mean Reads per Cell	860
Median Genes per Cell	219

Mismatch Infarcted zone

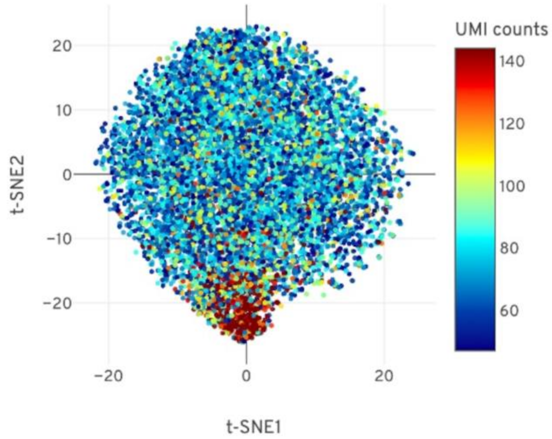

Estimated Number of Cells	74 040
Mean Reads per Cell	562
Median Genes per Cell	45

Match Border

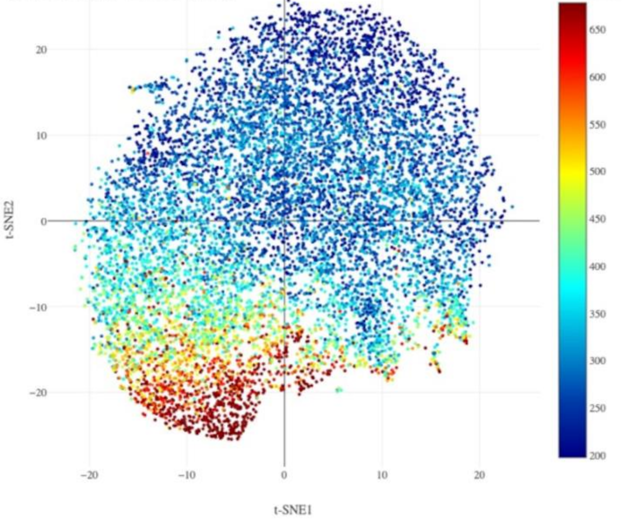

Estimated Numbero of Cells	80 216
Mean Reads per Cell	858
Median Genes per Cell	233

Mismatch Border

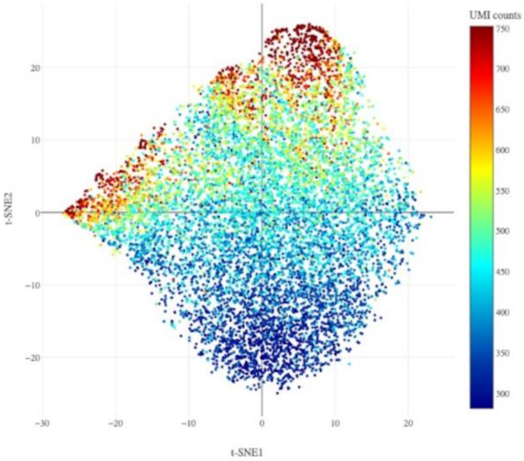

Estimated Numbero of Cells	78 451
Mean Reads per Cell	886
Median Genes per Cell	404

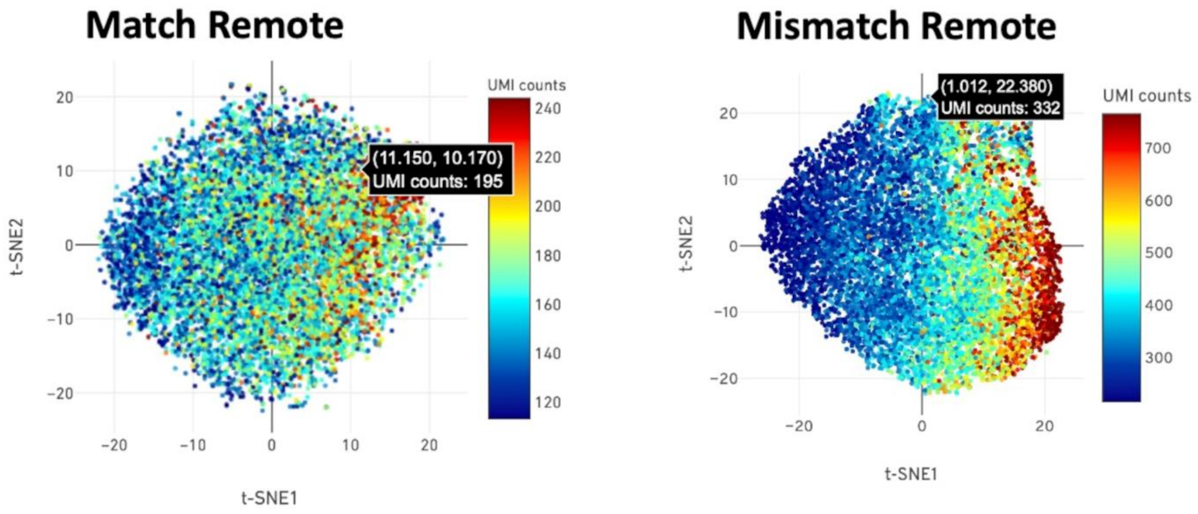

Estimated Numbero of Cells	80 590
Mean Reads per Cell	805
Median Genes per Cell	275

Estimated Numbero of Cells	82 381
Mean Reads per Cell	873
Median Genes per Cell	308

Supplementary Figure S6*. Quality control metrics of sequenced nuclei (10x Genomics snRNAseq) in infarcted, border, and remote zones of Match and Mismatch groups. Each dot represents sequenced nuclei in t-SNE projection of cell with color-coded UMI counts.*

Match Border

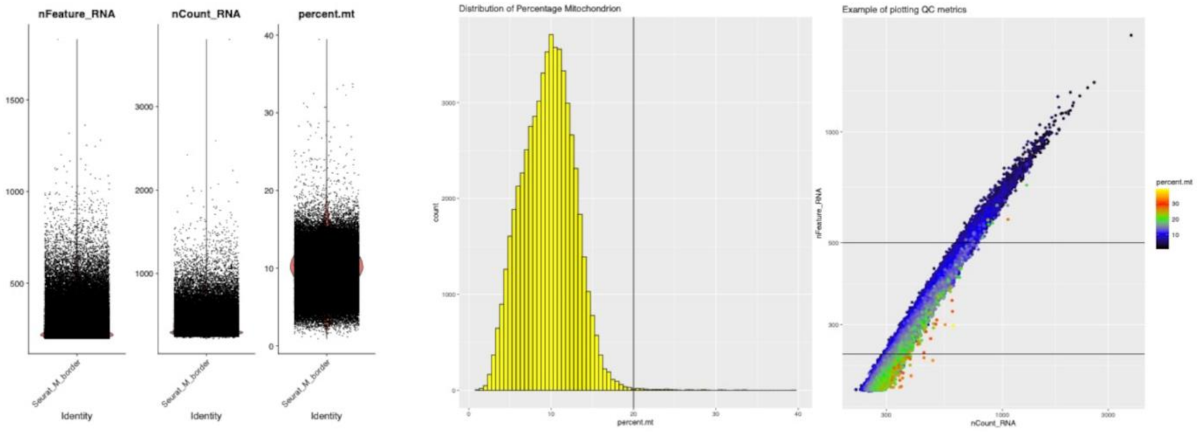

Mismatch Border

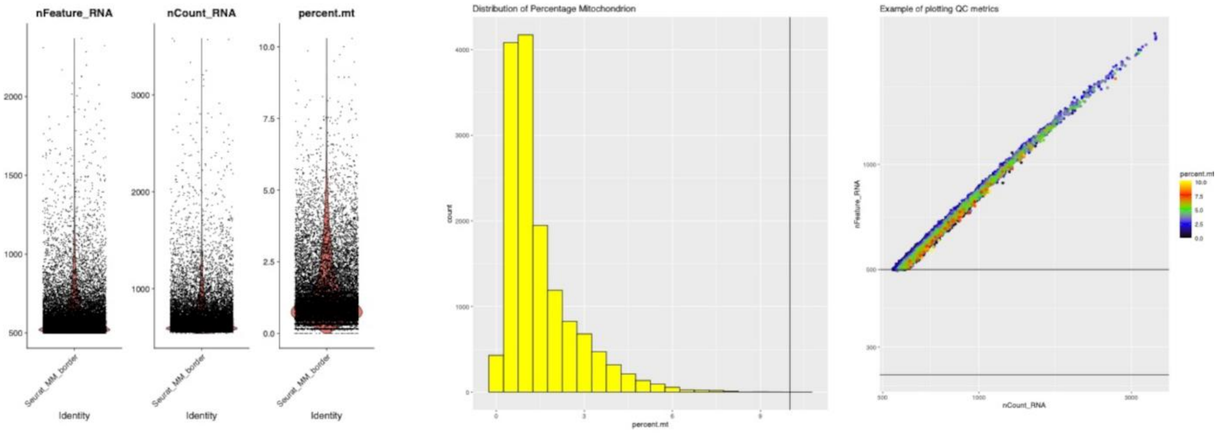

Match Remote

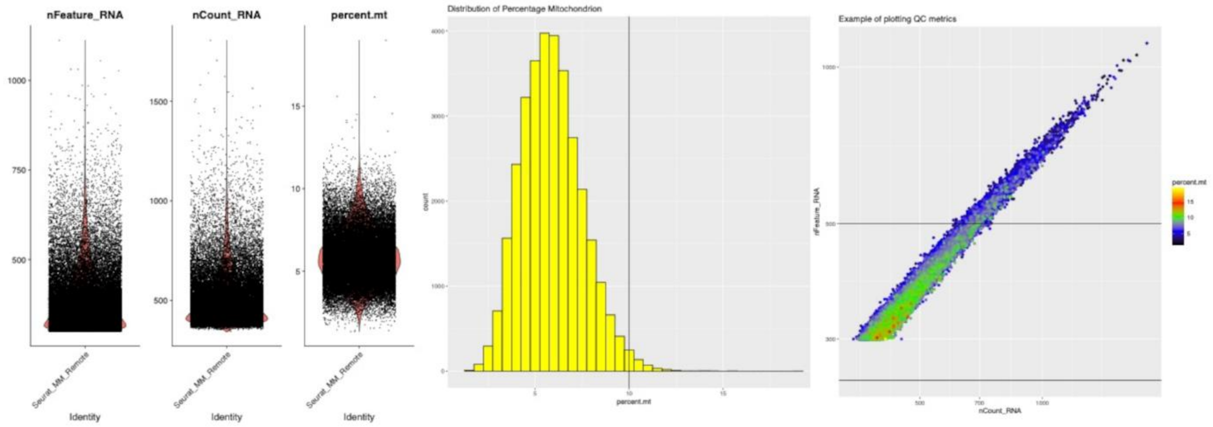

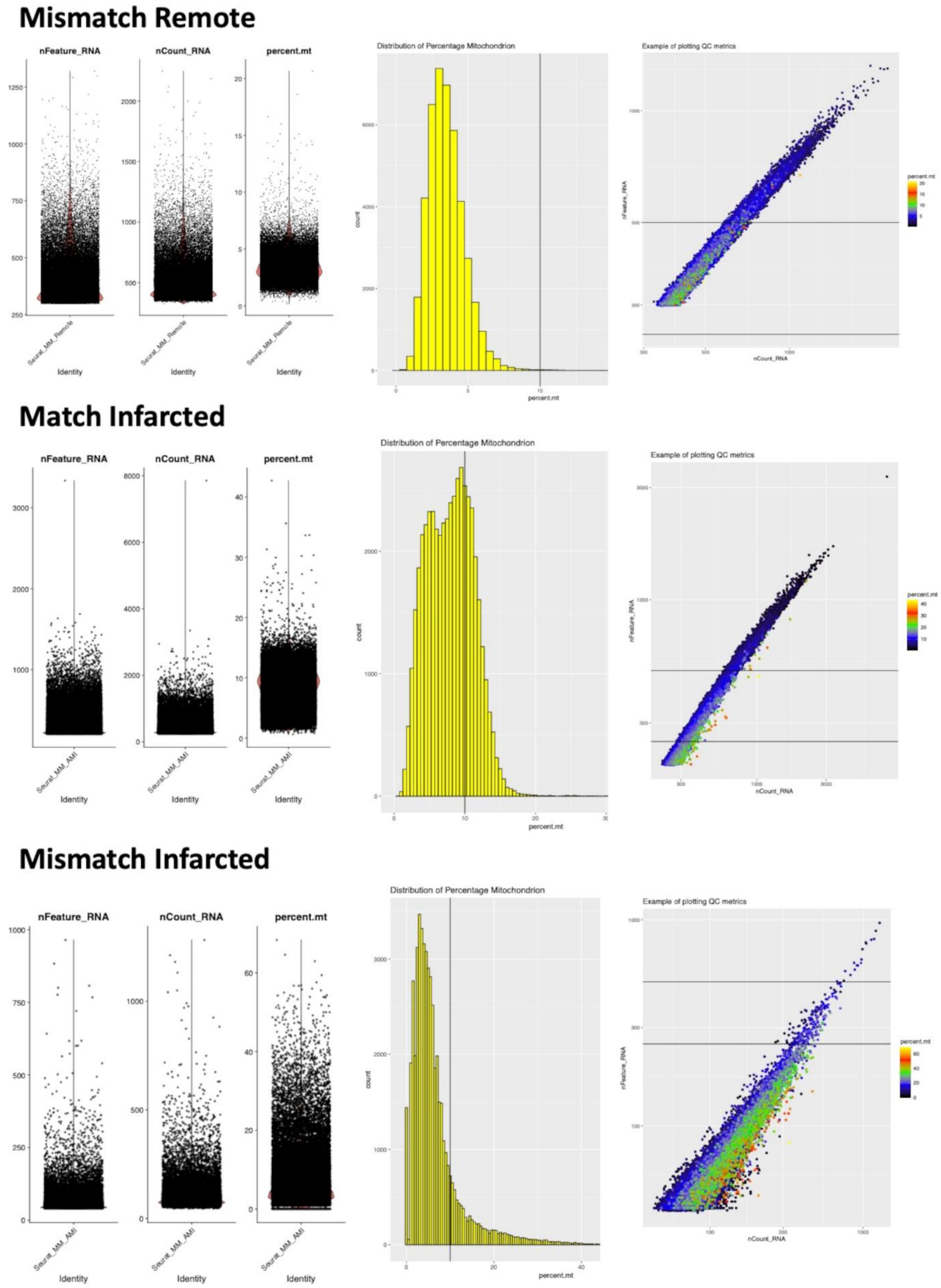

Supplementary Figure S7*. Data demonstrate a contribution of mitochondrial gene expression, suggesting the possibility of mitochondrial RNA adhering to nuclear membranes or being detectable through gene expression biochemistry. These genes were consequently excluded from downstream analyses.*

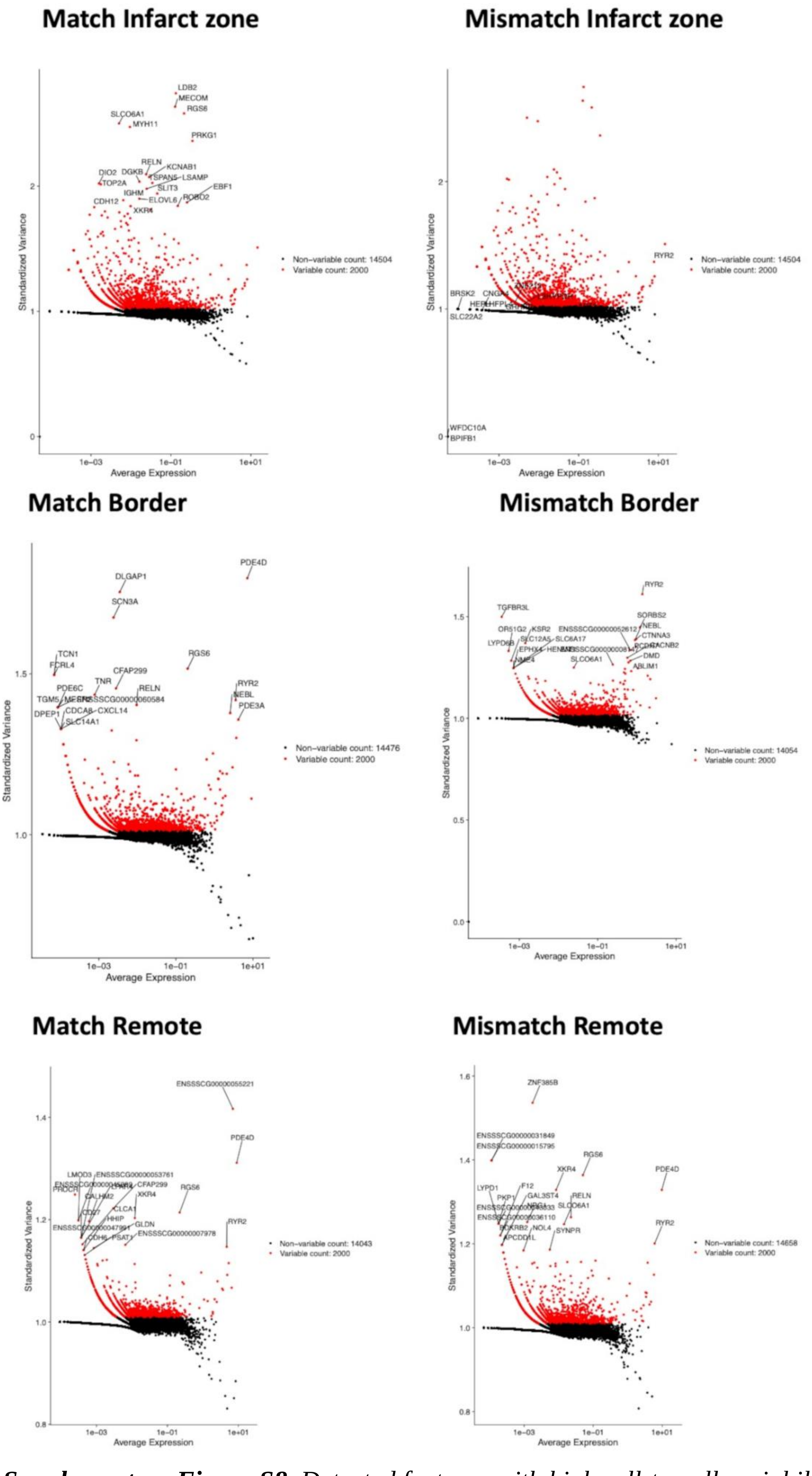

***Supplementary Figure S8**. Detected features with high cell-to-cell variability in the dataset. These genes were differentially expressed in some cell types and were thus of interest for further analyses.*

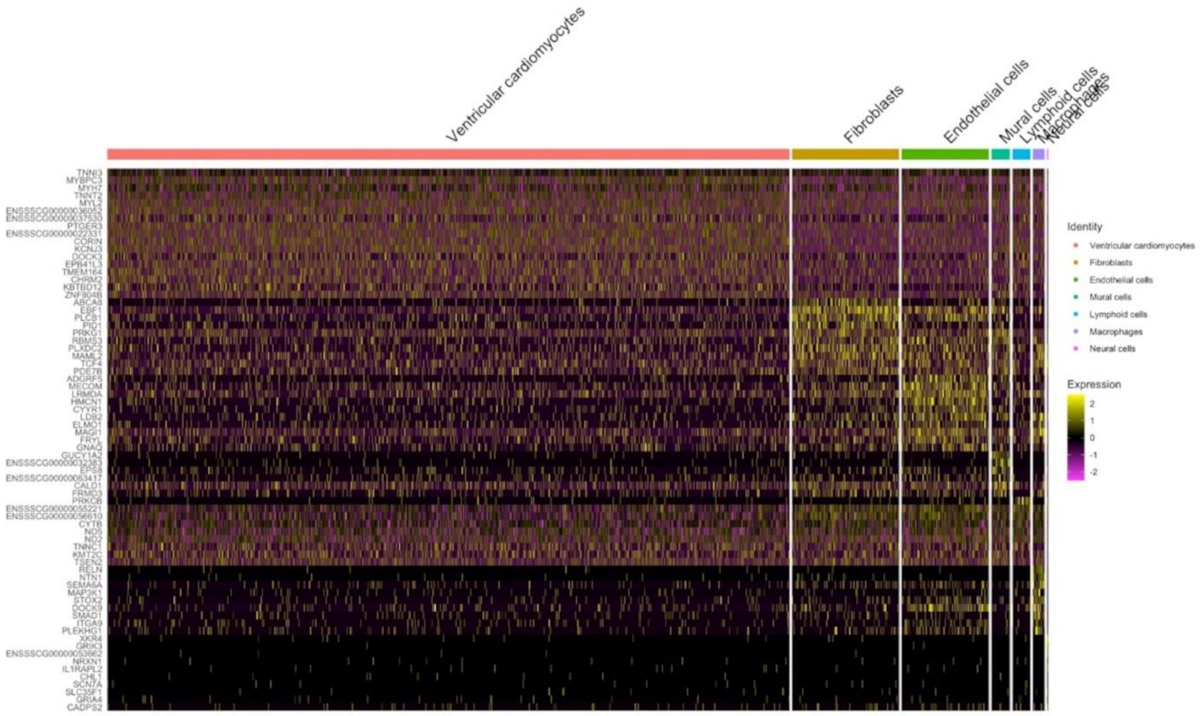

***Supplementary Figure S9**. Dimensionality reduction. Heatmap of significantly regulated genes according to Seurat integration analysis (FindMarkers function).*

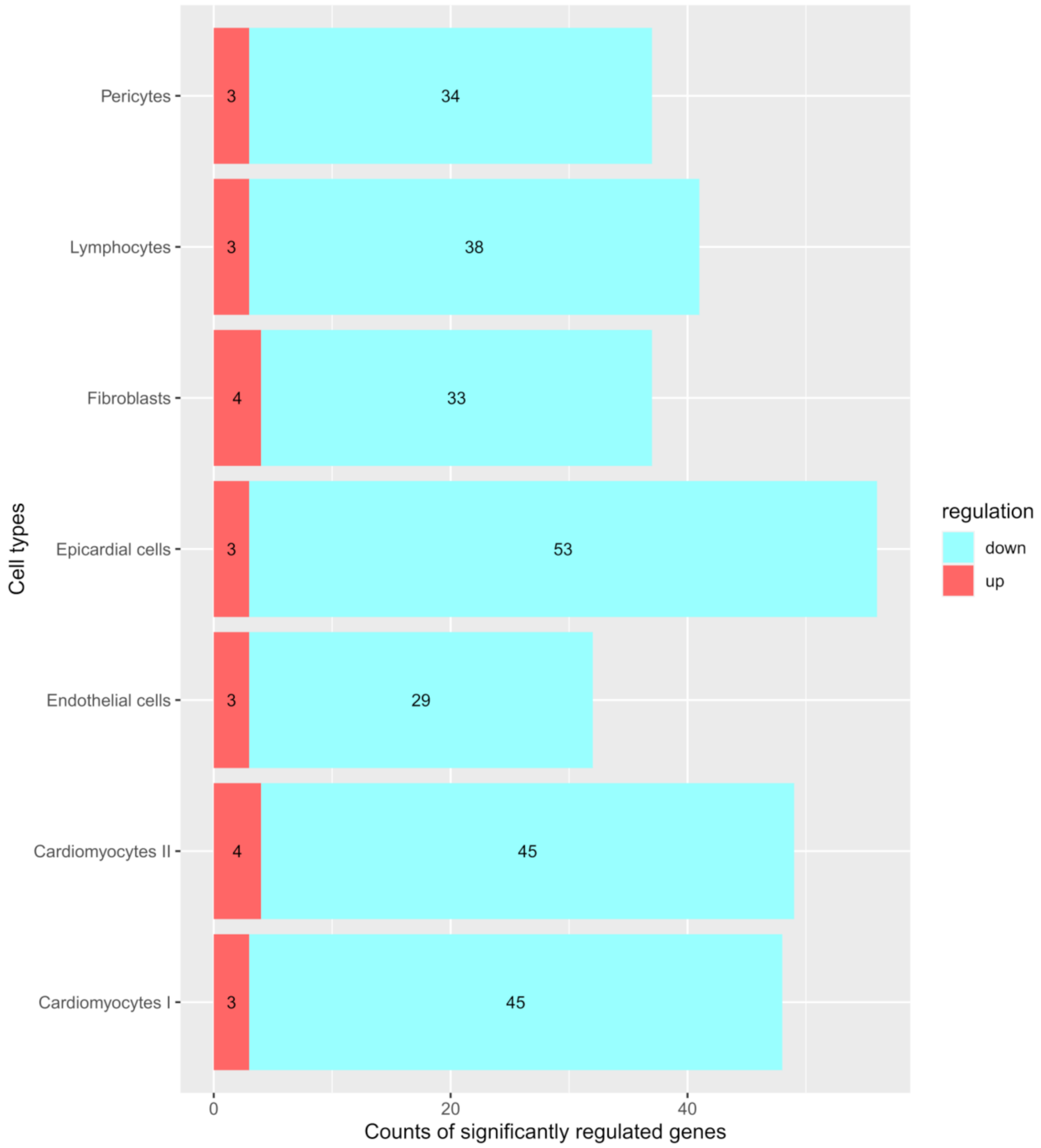

Supplementary Figure S10. *Numbers of differentially regulated genes in individual cell clusters between Match and Mismatch groups in the infarcted zone. Data were generated using the R-package MAST.*
